# How short-video usage influences college students’ daily lives: empirical evidence from sleep, self-perception, daily life conditions, and emotional state

**DOI:** 10.3389/fpsyg.2026.1855722

**Published:** 2026-10-02

**Authors:** Linmin Zhang, Huang Gelin, Xiujing Jiang

**Affiliations:** 1 Zhengzhou Institute of Science and Technology, Zhengzhou, China; 2 Research Center for Public Opinion and Cyber Information Security, Wenzhou University, Wenzhou, China

**Keywords:** college students, daily life conditions, self-perception, short-video usage, structure equation modeling

## Abstract

**Introduction:**

As the popularity of short-video apps rises among university students, issues have been identified linking problematic engagement to sleep, self-control, cognitive abilities, and emotion. However, the effects of bedtime procrastination and executive dysfunction on this relationship remain unknown.

**Methods:**

A cross-sectional study was conducted among 538 Chinese university students using an anonymous online survey. Pearson correlation analyses, observed variable path analysis, and indirect effect analyses were performed using the structural equation modeling framework.

**Results:**

Short-video addiction, bedtime procrastination, executive dysfunction, and emotional disturbance were found to be positively correlated. Within the structural model, executive dysfunction was found to correlate positively and significantly with emotional disturbance (*β* = 0.679, *p* < 0.001), whereas short-video addiction was found to be less strongly correlated (*β* = 0.133, *p* = 0.001). Furthermore, the correlation between bedtime procrastination and emotional disturbance was reduced to insignificance when executive dysfunction was added to the model (*β* = 0.018, *p* > 0.05). Model fit was inconsistent, showing adequate incremental fit but inadequate RMSEA fit.

**Discussion:**

It seems that executive dysfunctions play a significant role in emotional distress in problematic short-video users compared to bedtime procrastination. As this research was cross-sectional and self-report-based, causation cannot be established. Interventions should concentrate on the enhancement of executive functions and self-regulation abilities rather than screen time reduction only.

## 1 Introduction

Short-videos like Douyin, TikTok and Kuaishou have emerged as a significant part of the university students’ digital lives. Unlike traditional social media sites, which are mainly based on user-chosen networks or content, short videos integrate short AV content and persistent scrolling and recommendation systems (Montag et al., 2021). Such functions can contribute to extended viewing and have led to an increasing focus on understanding the potential behavioral and psychological correlates of problematic short video use by young adults (Cheng et al., 2023). But it is important to note that there is a difference between general short video use and problematic short video use (PSVU) or addictive short video use (ASVU). The idea of exposure, frequency or duration of viewing (as seen in general use) is different from that of compulsive and difficult-to-control engagement and watching that could intrude into other goals or activities (as seen in short video addiction (SVA) as explored in the present study). Analysis of recent studies has shown that problematic short video (SV) viewing is associated with sleep problems, self-regulation issues, cognition and emotional issues (Liu H. et al., 2025). The question that remains to be answered is then not whether these associations exist, but how these three areas of behavior, cognition, and emotion are interrelated when studied in the same analytical framework.

The study of problematic engagement can be informed by the design of short-video platforms as a credible context for behavior. Some of the signals (such as watch time, replays, likes, comments, and scrolling) can be used to continuously customize and tailor content, and the scrolling interface is low-friction, which reduces the difficulty of users switching between different items (Zhou, 2024). These may make withdrawal more challenging for certain users and foster reengagement, especially if digital activity is interfering with other activities, like academic work, socialization, or sleep. Such dynamics have been mentioned in the context of reinforcement processes in the field of behavioral addiction studies (Katsiroumpa et al., 2025). However, there is no direct test for algorithmic effects in the current cross-sectional study. Instead, the context of the platform features in the investigation of the relationship between problematic video engagement and self-regulation, sleep-related, cognitive, and emotional outcomes in college students (Zhai et al., 2024).

Bedtime procrastination represents one plausible behavioral pathway linking problematic short-video engagement with poorer well-being. Delays that are intentional and for no external reason (Kroese et al., 2014). This could be even more of an issue for university students, especially since they often have flexible schedules, a heavy workload, and lots of time in front of their phones at night. The utility of the short video platforms for this behavior may be due to the constant availability of content, which can cause a disruption of intended sleep schedules and increase sleep disengagement at bedtime. There have been previous studies that have associated bedtime procrastination and smartphone use problems with reduced sleep among university students (Zhang and Wu, 2020), (Ye et al., 2022). Sleep-related behaviors, however, such as procrastination at bedtime are crucial, but may not fully account for the relationship between problematic short video use and emotional distress.

This second, cognitively based explanation is called executive dysfunction. Executive functions, such as inhibitory control, working memory, attentional regulation and cognitive flexibility, underlie goal-directed behavior and resisting distraction (García-Maza et al., 2025). The following difficulties in these domains can be seen as signs of problems with attention, recall of intended actions, inhibiting responses, and shifting attention when moving between tasks. In the past, high engagement with short videos was found to be related to decreased attention-related functioning, so that executive regulation is a pertinent construct in this regard (Yan et al., 2024). Importantly, there is a relationship between executive dysfunction and bedtime procrastination, but they are conceptually separate. The main theoretical issue is whether bedtime procrastination has an independent relationship to emotional distress when executive-regulatory difficulties are examined concurrently or if executive-regulatory difficulties account for more of the observed relationship. These pathways are important to distinguish because a sleep-related explanation means a different interpretation of digital vulnerability than a cognitive-regulatory implication because they lead to different interpretations of digital vulnerability (García-Canalejas et al., 2025).

In addition, a regulatory aspect of this framework is given by self-control. Self-control is the ability to delay gratification, control behavior in the face of competing rewards, and maintain longer-term objectives. Students who have high self-regulatory ability might have more control over their screen time, be less likely to be involved in screens at the intended bedtime, and more likely to follow their academic routines, while low self-regulatory ability may be linked to higher susceptibility to problematic engagement (Tangney et al., 2018). For the present analysis, self-control items that used positive wording were reverse coded, so that higher composite scores indicate more of a self-control deficit; thus, the expression self-control deficit is used when referring to the score analyzed. A relevant population is also college students since there are some significant demands in college, including autonomy and self-regulation, which are part of emerging adulthood. Understanding the intersection between problematic digital engagement and cognitive and emotional functioning might be more relevant in this developmental context (Meier and Reinecke, 2021; Kabasakal and Çelik, 2025).

With the Chinese context, these relationships are significant because the platforms of Douyin and similar short video platforms play a significant role in the daily digital life of people. Meanwhile, the digital engagement of students might differ by socioeconomic and regional contexts (Zhu C. et al., 2024). Thus, the present study emphasizes the Chinese college students, and regional differences are viewed as context rather than as the theoretical comparison. This distinction is relevant as the analytic sample is focused on specific areas of interest and is not intended to make general inferences about interprovincial differences. The focus instead is to explore the relationship among problematic viewing of short videos, self-regulation, bedtime habits, executive functioning and emotional distress in the sample of observed students (Yang et al., 2025).

Based on this, this study explored the relationship between short-video addiction and self-control deficit, bedtime procrastination, executive dysfunction and emotional distress in a cross-sectional sample of 538 anonymous Chinese college students through an online questionnaire. In the primary analysis, the interrelationships among these constructs were assessed using standardized composite scores, especially the relative contributions of bedtime procrastination and executive dysfunction to emotional distress. The paths proposed are modeled as associations, rather than as evidence of a chronological process, and they are compared within the same model. The purpose of this approach is to determine if sleep-related delay and executive-regulatory problems separately or together (Sun et al., 2026) contribute unique explanatory information.

The study will make three major contributions. First, it links short-video addiction, self-control deficit, and bedtime procrastination, executive dysfunction and emotional distress, but these different domains are not studied separately. Second, it directly examines the relative association of bedtime procrastination and executive dysfunction to emotional distress, which in turn allows for the differentiation of the sleep-related and cognitive-regulatory explanations of problematic short-video engagement. Third, it provides evidence from a sample of Chinese students in the university setting but importantly acknowledges that a cross-sectional, self-report design is limited. In keeping with this design, all of the structural and indirect pathways are considered to be associations and not necessarily a temporal or causal pathway. The study thus examines the following research questions: Is there a correlation between problematic short video engagement and increased bedtime procrastination, self-control deficit, executive dysfunction, and emotional distress? Does there exist a stronger independent correlation between emotional distress and self-control deficit or bedtime procrastination when both are controlled for?

## 2 Literature review

### 2.1 Theoretical foundations

The addiction to short videos has become more of a multidimensional problematic engagement, rather than just the amount of time spent using it. Preoccupation and Persistence, Withdrawal and Tolerance, Harms and Disability, and Deception and Escape were measured using the Short-Video Dependence Scale (SVDS) (Jiang A. et al., 2025) in a large sample of 16,038 participants. These dimensions differ from brief screen time or normal viewing of short videos in that they highlight lack of control, continued use, and consequences of use. It’s important that this multidimensional approach is used since problematic engagement during short videos can be analyzed independently from mere time spent watching. Self-control failure is also defined as “media use that interferes with important behaviors like academic study or sleep” and is seen as more informative than duration for negative outcomes, as described by (Miedzobrodzka et al., 2024). From this perspective, problematic use can be understood as a self-regulatory conflict in which immediate digital rewards compete with longer-term academic, sleep, or personal goals.

Problematic short-video engagement may also be related to self-perception. Research studies based on self-discrepancy and self-perception theories have been shown to correlate idealized online self-presentation, short-video engagement with self-identity, self-esteem, appearance anxiety, and changes in digital self-presentation (Kim and Sundar, 2012; Zhu R. et al., 2024; Zhu J. et al., 2024). The results of these studies offer background information to support the investigation of self-regulatory and self-perceptual processes in the present study, where the specific regulatory construct investigated is self-control deficit.

### 2.2 Empirical literature

#### 2.2.1 Impact on sleep quality and physical health

Student problem short-video use correlates are among the most discussed issues in the literature. Three hundred and fifty-three (25.2%) of students with clinically relevant sleep disturbance were found, based on regional evidence from Henan. Sleep was an important domain of concern in this population. Zhao and Kou (2024) found that the relationship between problematic short-video use and sleep might be mediated by physical activity and procrastination, indicating that the relationship between problematic short-video use and sleep may be a chain mediation pattern that is not solely as a result of exposure to short videos. Further supporting the relationship between problematic engagement and sleep outcomes, Al-Adamat and Bani Khalid (2026) found that there was a significant variation in sleep quality explained by indicators and daily internet use related to internet addiction.

The present study does not explicitly test the algorithmic or physiological processes, but experimental work (Feng et al., 2026) indicates that various recommendation contexts could have different sleep-quality outcomes. Similarly, there was a correlation between problematic TikTok use and daytime drowsiness among adolescents (Bilali et al., 2025). As the present study does not index physiological sleep mechanisms, this literature is largely used as arguments in support of the plausibility of sleep-delay behavior as a correlate of sleep problems with short video use.

#### 2.2.2 Cognitive functioning and academic performance

At the same time, there has been a growing concern about the negative consequences of engaging in problematic short video use, including cognitive and academic problems, especially attention and executive control. Some other avenues of potential contribution to poorer academic outcomes than time displacement were suggested in another study (Zhang and Zeng, 2024) which found that academic anxiety was a mediator between addictive digital behaviors and poorer academic outcomes. Other studies mentioned in the manuscript link low attentional control, attentional fragmentation, and problems to paying attention with problematic short video watching (Xie et al., 2023; Chen et al., 2023). In terms of resources, extended or unregulated involvement has also been linked to burnout, decreased mindfulness, and decreased learning engagement (Mao and Liao, 2025; Jia, 2025; Li et al., 2024). This literature, when put together, indicates that impaired executive function (e.g., inhibitory control, working memory, and cognitive flexibility) may be a cognitive-regulatory correlate of problematic short-video use. Importantly, executive dysfunction may overlap with, but is not identical to, bedtime procrastination; examining both simultaneously can clarify whether emotional distress is more closely associated with behavioral sleep delay or broader cognitive-regulatory difficulties.

However, in spite of such risks, internal mindsets and behavioral traps have a moderating effect on the influence of short videos on academic life. Wang (2025) introduces a two-fold impact model, as the platform provides them with micro-learning opportunities; the high speed of delivery trains the brain to act in a superficial way instead of deep learning. Similarly, Jiang Y. et al. (2025) model to illustrate the idea that losing track of time is not one of the cognitive failures perceiving time, but a behavioral trap formed by the auto-scrolling that has resulted in colossal usage-reality gaps. In order to counteract those effects, Qi et al. (2024) recognized the following as the final protection shields: growth mindset and sense of agency; students who strongly believe in their own development are able to turn the exposure of digital sources into motivation. Lastly, according to a meta-analysis by Wang et al. (2025) there is scant mention of pre-sleep device usage as a leading behavioral antecedent to a national public health emergency in Chinese universities where academic stress and digital dependence go hand in hand to worsen a 25.7% sleep disorder prevalence, especially in high-competition educational institutions such as Henan.

#### 2.2.3 Psychological well-being and self-perception

Another important result that is included in the short video literature is psychological welfare. Jiang and Yoo (2024) connected short-form video addiction to poorer sleep and found that social anxiety was an important hypothesized intermediate correlate of problematic engagement, indicating that affective/relational processes may be co-occurring with problematic engagement. There have also been some related studies that have linked notifications, peer comparison, and increased platform engagement to an increase in stress and social anxiety (Saleem et al., 2026). The broader conceptual explanation offered by Zhan and Zhu (2025) is the I-PACE framework, which focuses on the interaction between individuals’ behavioral control, cognition, affective responses and problematic use.

Other mechanisms that are assessed indirectly in the present study, but described in the literature, include, but are not limited to, experiential avoidance, loneliness, and neurobiological reward processes, all of which are considered to be contextual rather than tested mechanisms (Ye et al., 2023). The present analysis focuses mainly on emotional distress in relation to short-video addiction, the procrastination of night time, deficit of self-control and executive dysfunction.

#### 2.2.4 Regional and socioeconomic moderators (Zhejiang vs. Henan)

The pattern of digital engagement in China may differ by region and socioeconomic factors, but there is no evidence that such a pattern is uniform across all provinces. The study by Shao et al. (2018) found differences between regions, and Zhang and Zhu (2025) highlighted shifts in digital gratification in underdeveloped regions, suggesting that the nature and impacts of digital engagement can be shaped by economic and social conditions.

One socioeconomic and technological boundary condition can be digital self-efficacy, which was proposed by Xie et al. (2025) as an explanation of the purposefulness of use or passivity of use of short-video platforms. However, there is always the possibility of the physiological exertion of the medium in this active agency. Experimental research by Chiossi et al. (2023) also suggests that the rapid shift of contexts in short-form video contexts can affect prospective memory, which may also be relevant in a regional context and offers a cognitive explanation. The report by Miao and Pan (2025) and Chen et al. (2026) also suggested that the younger and less developed areas are more dependent yet, this finding cannot be applied to make a blanket statement that one province is more vulnerable than another. The present sample is heavily skewed in Henan and can therefore only be used as a context for the regional results and not as a basis for a balanced comparison of Henan and Zhejiang.

### 2.3 Synthesis and research gap

The reviewed literature actually revealed significant results related to the correlation between problematic short video use and sleep-related behavior, self-control, executive function and emotional well-being and hence, the gap is not the absence of research on short video addiction per se. The more specific unresolved question is the relationship between bedtime procrastination and executive dysfunction and emotional distress from a single analytical framework, especially when these are conceptual and empirically similar to self-regulatory processes. Thus, in the present study, the relative associations of bedtime procrastination and executive dysfunction with emotional distress are examined, but the proposed pathways are conceptual and not temporal or causal in nature.

## 3 Methodology

### 3.1 Study design and research setting

This study adopted a cross-sectional survey design to explore the relations between short video addiction, bedtime procrastination, self-control deficit, executive dysfunction and emotional distress among college students in China. This design was chosen as it was intended to describe the co-occurrence and statistical relationships between behavioral, cognitive-regulatory, and emotional difficulties from a shared sample of students. All exposure, mediator and outcome variables were measured at the same time and therefore the analysis of the data was seen as associations over time and indirect-effect patterns, instead of temporal ordering and causation.

The participants lived in several parts of China, mostly in Henan Province with a smaller sample from Zhejiang and other provinces. The study was thus not a comparative design between Henan and Zhejiang, which was balanced. Geographic information was not used in the primary analyses of this study, but was retained for descriptive profiling and secondary exploratory comparisons of individual-level relationships among the five main constructs of the study.

The workflow involves preparing data, evaluating the measurement, a single primary observed-variable path model, indirect-effect analyses with bootstrapping for each of the secondary models, and a smaller number of robustness analyses. Primary methodology does not include exploratory machine-learning, clustering, network and invalidated index-development procedures.

### 3.2 Data collection tool and procedure

The data collection tool utilized was an online anonymous questionnaire conducted via Sojump. The participants for the research were selected from Chinese college students familiar with short-video platforms using convenience sampling. Prior to taking the survey questions, the respondents were provided with information about the academic nature of the study, its voluntariness, anonymity of responses, and their right to terminate their participation in the process at any point in time. Participants were asked to recall their behavior during the preceding month, especially the one prior to survey completion (see Table 1).

**Table 1:** Structure of the survey instrument.

Part	Section	Domain of measurement	Key indicators/variables
1	Demographics	Baseline Characteristics	Gender, age, academic year, financial status, and daily usage time
2	Usage Experience	Short-Video Addiction	Withdrawal symptoms, loss of control, and functional impairment
3	Sleep Habits	Bedtime Procrastination	Intentional sleep delay and nighttime media distraction
4	Self-Description	Self-Control	Trait-level self-regulation and impulse management (incl. Reverse-coded items)
5	Life Conditions	Executive Functioning	Behavioral indicators of inhibition, working memory, and cognitive flexibility
6	Emotional State	Emotional Distress	Physiological and psychological distress symptoms (Likert-based)

In the course of this research, short-video platforms were considered applications that employ a “scroll” method of presenting short audiovisual content, including platforms such as Douyin, TikTok, and Kuaishou. The anonymous questionnaire comprised demographic data, patterns of short-video exposure, and five main psychological/behavioral constructs, such as short-video addiction (SVA), bedtime procrastination (BP), self-control deficit (SC), executive dysfunction (ED), and emotional distress (Emo). Psychometric measures were assessed using a 5-point Likert scale.

Items of self-control were reverse-coded before making the composites, to ensure consistency in the direction of the self-control variable in the composite, with the vulnerability-oriented interpretation of the other variables. Thus, self-control deficit is used as a term throughout the revised manuscript; higher scores on the SC would be a lower, rather than higher, self-regulatory control. To maintain the original 1–5 metric and for ease of interpretation across constructs, mean aggregation was performed.

### 3.3 Sample, eligibility, and analytic dataset

The final analytic dataset contained 538 valid student responses. The responses were kept after applying the study eligibility criteria and after preparing the variables to be used in the planned analyses after quality control. Demographic and regional characteristics were reported descriptively, and were not used to make claims of prevalence or province level effects because of geographic and academic concentration of the sample.

The data were gathered through an anonymous online questionnaire (Sojump) in late December 2025. This survey focused on college students living in China, most of them came from Henan province and Zhejiang province, a small number from other provinces. The survey was self-selected and respondents answered the questionnaire for their experiences in the past month. Prior to analysis, data were screened for data quality, such as by using an attention-check item and a criterion for completion time designed to detect inattentive or implausibly fast answers. After the procedures of screening, the analytic sample consisted of 538 valid participants. Data quality criteria were prespecified and all eligible responses that met the criteria were used in the analysis. There was no *a priori* power calculation.

### 3.4 Ethical considerations

This study was approved by the Ethics Committee of Wenzhou University. The study was anonymous and voluntary. Participants were given information about the purpose of the study, as well as the fact that participation was voluntary, their responses would be kept confidential, and the data they provided would be used for academic research. The ethical approval number was 001. Participants were only allowed to carry on with the questionnaire once they gave informed consent. The survey did not gather any personally identifying information.

### 3.5 Data preprocessing and quality control

Raw survey data was downloaded from Sojump and then carefully reviewed for statistical analysis. The variables were checked for their formats, standardized, and inconsistencies in geographic information were corrected and all psychometric items were coded in the correct direction during data preparation. The self-control items were reverse coded to ensure that higher scores on each of the vulnerability-oriented constructs reflected greater self-control deficit, or greater regulatory difficulty.

The screening procedures built into the survey were utilized to determine data quality. Responses that did not satisfy the embedded attention-check requirement or that showed implausibly rapid questionnaire completion were excluded before construction of the analytic dataset. Data quality criteria were established and only responses that met these criteria were retained, yielding an analytic sample of 538 participants.

After screening, items for each theoretical construct were classified based on the pre-established measurement structure. The composite scores for the domains of Short-Video Addiction (SVA), Bedtime Procrastination (BP) (Chen et al., 2026), Self-Control Deficit (SC), Executive Dysfunction (ED) and Emotional Distress (Emo) were derived by summing up the items of each domain using an arithmetic mean. The number of responses was converted to a 1–5 response metric using mean aggregation for ease of interpretation and comparison across constructs. Usable responses were used for each scale to compute a composite score, and observations with fewer than sufficient responses to form a scale score were not included in analyses that involved the corresponding scale.

The coding of all composite variables was then checked for errors, implausible values, distributional characteristics, and consistency before inferential and structural analyses were performed.

### 3.6 Variable construction and scoring

#### 3.6.1 Demographic variables

Demographic variables were gender, academic year, daily short video viewing, length of the month’s living expenses, and geographic location. These variables served mainly as descriptive of the sample and as (theoretically relevant) covariates or exploratory moderators. Regional comparisons were made as a second priority as the sample was not balanced over provinces.

#### 3.6.2 Psychological and behavioral composite scores

Items were clustered based on the conceptual domains of each item that was pre-specified. The arithmetic means were computed row-wise for SVA and BP, SC, ED, and emotional distress, keeping the original 1–5 response scale. This method gives directly interpretable composite scores without artificially extending the score range because of varying numbers of items on the constructs (He et al., 2023). An increase in the scores of SVA, BP, ED, and emotional-distress scores were associated with increased severity. Items in the test were reverse scored to positive items and higher scores in the self-control scale meant a higher degree of self-control deficit.

### 3.7 Measurement quality assessment

The quality of the multi-item measures was assessed before analysis of the structural relationships between the study variables. The internal consistency, factorability, preliminary factor structure and empirical overlapping between the main constructs were examined (He et al., 2023). The reliability and interpretable of the composite scores were determined using these diagnostics to ensure reliable and interpretable scores for further correlation, regression, and path analyses.

#### 3.7.1 Internal consistency

Each construct consisted of more than one item, and internal consistency reliability was evaluated for each of these using Cronbach’s alpha. Those coefficients greater than around 0.70 were considered to be good for the purposes of group level analysis. Reliability estimates were reported for each construct instead of being based on a single overall coefficient for conceptually different domains.

#### 3.7.2 Factorability and preliminary factor structure

The Kaiser–Meyer–Olkin (KMO) measure was used for the sampling adequacy for the factor analysis. As a preliminary evaluation, exploratory factor analysis (EFA) was then performed at the item level to determine if the general pattern of covariance observed broadly matched the theoretically posited domains of Short-Video Addiction, Bedtime Procrastination, Self-Control Deficit, Executive Dysfunction, and Emotional Distress. Items with factor loadings around 0.40 or higher were considered to be substantively interpretable, and items with significant cross loadings were reviewed for possible conceptually similar items (He et al., 2023).

The factor analysis results were considered preliminary evidence of the organization of the measurement items in the study constructs, since the constructs were expected to be correlated. The overlap between Self-Control Deficit and Executive Dysfunction (SCC) was of special interest since this construct significantly correlated in later analysis. For this reason the EFA was mainly conducted as a diagnostic tool to test the structure of the measurement, and conclusion regarding the discriminant validity was determined independently based on more valid construct-level criteria.

#### 3.7.3 Discriminant validity

Given the considerable conceptual and empirical overlap between Self-Control Deficit (SC) and Executive Dysfunction (ED), this was handled separately from multicollinearity. Therefore, variance inflation factors (VIFs) were only used to check for potential instability of regression and path coefficients and were not interpreted as evidence that the psychological constructs were empirically distinct dimensions.

Careful consideration was devoted to the difference between SC and ED since these two are closely associated. Thus, caution was taken in the interpretation of the evidence derived from the exploratory factor structure, and discriminant validity was not taken for granted based on reasonable reliability or VIF statistics. A more precise evaluation needs to be conducted at the item level, for example, by calculating the heterotrait – monotrait ratio (HTMT) and, if applicable, confirmatory factor analysis (CFA) using the composite reliability and average variance extracted (AVE).

The difference between Self-Control Deficit and Executive Dysfunction should be considered as theoretically suggested but not empirically confirmed until the rest of the measurement diagnostics are finished.

#### 3.7.4 Common-method variance diagnostic

Common-method variance was considered as all the main variables were derived from the same self-administered questionnaire. To get an initial sense of the existence of a single factor with dominant covariance structure, Harman’s single factor diagnostic was employed. The cautious interpretation of the results of this diagnostic was warranted because only a single cross-sectional self-report study was performed so that the potential for shared-method variance was not excluded.

### 3.8 Descriptive statistics and preliminary analyses

Before performing the multivariable modeling, preliminary analyses were performed to describe the study sample, and the distribution and bivariate relationships of the key study variables were examined. These analyses gave an overview of the sample composition, central tendencies, variability and distributional characteristics of the composite measures and were employed to detect any characteristics of the data that might impact the subsequent statistical interpretation.

#### 3.8.1 Sample profiling and distributional assessment

Data on participant characteristics were presented in the form of frequencies and percentages. Means, standard deviations and 95% confidence intervals were used to summarize composite scores. Histograms and skewness and Kurtosis were used to explore distributional properties to determine whether there was significant deviation from approximate normality, and to guide use and interpretation of parametric procedures.

#### 3.8.2 Bivariate associations

Before multivariable modeling, bivariate relationships between SVA, BP, SC, ED, and emotional distress were described in terms of their magnitude and direction by calculating Pearson product–moment correlation coefficients. Correlations were used descriptively as relationships and should not be interpreted as being directional. The high correlation of SC with ED has been of particular concern here, due to the overlap of the two constructs and the interpretation of the coefficients.

#### 3.8.3 Multicollinearity diagnostics

For the predictors included in regression-based models, variance inflation factors (VIFs) were computed to assess the potential for multicollinearity to increase the standard errors of the regression coefficients and/or to cause coefficient instability. Any coefficient value below a conventional concern threshold was interpreted to suggest severe coefficient level multicollinearity was not a concern. In the assessment of discriminant validity, the results of the VIF test were excluded.

#### 3.8.4 Exploratory demographic heterogeneity

Independent-samples t-tests were used to compare gender differences on the key constructs, with variance adjusted estimates used when the assumption of homogeneity of variance was violated. The differences between academic years were compared by one-way analysis of variance (ANOVA) and Tukey-adjusted *post hoc* comparisons were conducted when the omnibus test was statistically significant. A number of effect sizes were reported in addition to *p*-values to differentiate group difference from significance.

### 3.9 Primary observed-variable structural path model

The main model analyzed by the multivariable analysis was an observed-variable path model estimated within a structural equation modeling (SEM) framework. Standardized composite scores were used for the five main constructs instead of latent factors. This is significant because this distinction does not mean the model is a latent variable measurement model, but rather it’s a relation among observed scale composites.

The revised structural framework was organized around one central question: whether bedtime procrastination retained an independent association with emotional distress after executive dysfunction and short-video addiction were considered simultaneously. Short video addiction, self-control deficit was selected as the predictors of bedtime procrastination. The emotional distress was defined as a function of short-video addiction and executive dysfunction and bedtime procrastination. To account for the theoretical and empirical overlap, the covariance between self-control deficit and executive dysfunction was freely estimated.

#### 3.9.1 Model specification

The path coefficients were estimated using composite scores that had been standardized for comparison. Maximum-likelihood procedures were used to estimate the path model. Standardized coefficients, standard errors, *p*-values, and confidence intervals were reported for the principal structural paths.

#### 3.9.2 Model fit evaluation

The Comparative Fit Index (CFI), Tucker–Lewis Index (TLI), and Root Mean Square Error of Approximation (RMSEA) were used to assess the model fit. The higher the CFI and TLI are, the more the model fits the data, and higher values around 0.90 or higher were considered acceptable incremental fit, and higher values around 0.95 or higher was considered a stronger fit. RMSEA values < ~0.08 were considered acceptable absolute fit and values < ~0.05 were considered close fit. The indices were read together, and one fit index was not considered to be a good model if it showed a significant misfit.

#### 3.9.3 Interpretation of structural paths

The focus was mainly the relative size and statistical significance of the paths to emotional distress. Specifically, after the executive dysfunction was added to the model, the association of the bedtime procrastination and emotional distress was evaluated. As the data were cross-sectional, paths that were statistically significant were interpreted as conditional associations, rather than causal effects or evidence that one construct came before another.

### 3.10 Indirect-effect analyses

Two plausible explanatory paths were examined between short video addiction and emotional distress, without ordering the pathways of BP and ED, using two separate single-mediator indirect-effect models. This is the first revision of a former chain formulation where executive dysfunction and bed time procrastination were considered a fixed-sequence in chronological order.

#### 3.10.1 Bedtime-procrastination indirect-effect model

The first model was an indirect model that tested the effect of short video addiction on emotional distress via a mediator (bedtime procrastination). The model estimated the SVA-to-BP path, the BP-to-emotional-distress path, the direct association of SVA with emotional distress after adding BP, and the product term representing the indirect effect.

#### 3.10.2 Executive-dysfunction indirect-effect model

The second model investigated the indirect relationship between short video addiction and emotional distress via the mediation of executive dysfunction (SVA → ED → emotional distress). This model accounted for a cognitive-regulatory pathway without the need for BP to precede ED or ED to precede BP.

#### 3.10.3 Bootstrap inference

Indirect effects were evaluated using bootstrap resampling with 2,000 resamples to obtain bias-corrected 95% confidence intervals. An indirect effect was deemed statistically supported when the interval included no zero. The direct, indirect and total effects were reported separately. When this term was retained for continuity with the conventional statistical usage, it was understood as an indirect association (cross-sectional) and not as a temporal or causal mechanism.

### 3.11 Secondary and robustness analyses

#### 3.11.1 Moderation by monthly living expenses

A secondary moderation analysis was performed to determine whether the relationship between SHV addiction and emotional distress was moderated by monthly living expenses. Nonessential collinearity was reduced and the continuous predictors were standardized prior to creating the interaction term to ensure the interpretability of the analysis. The interaction coefficient and not the subgroup means were used to see if the strength of association varied across levels of monthly expenses.

#### 3.11.2 Exploratory thrill-seeking moderation

To examine the effect of thrill seeking on the relationship between short-video addiction and executive dysfunction, another exploratory moderation model was tested. The analysis was not included in the central structural framework but rather used as a second level personality-based analysis. In order to be able to interpret any interaction terms, it was also included in the same regression model as main effects and interpreted with caution, since it was not the main hypothesis of the study.

#### 3.11.3 Robust regression diagnostics

Heteroskedasticity-consistent HC3 standard errors were applied in regression-based secondary analyses, as appropriate. Major departures from linear-model assumptions were explored by looking at residual plots, LOWESS curves, leverage, and influence diagnostics to locate observations that have a disproportionate influence on the estimated coefficients.

#### 3.11.4 Hierarchical regression sensitivity analysis

To test for the substantive pattern in emotional distress, hierarchical regression analysis was used as a sensitivity analysis to explore whether the substantive pattern persisted when using a different regression specification. The order of block entry was demographic covariate, short-video addiction, and the main cognitive-regulatory variables. The incremental contribution of each block was assessed by calculating changes in explained variance (Delta R-squared), standardized coefficients and significance tests. The regression analysis was not considered as an alternative competing theory but rather was used to support the model.

### 3.12 Statistical reporting and reproducibility

All of the statistical tests were two-tailed and the level of statistical significance was set at 0.05. Effect estimates with associated *p*-values and 95% confidence intervals, if available. The primary inferential models were the observed-variable structural path model and separate indirect-effect models for Bedtime Procrastination (BP) and Executive Dysfunction (ED). Demographic comparisons, moderation analyses and hierarchical regression were interpreted as additional analyses for providing information on the differences among subgroups, possible effect modification and robustness of the main association.

The data was prepared and statistical analyses conducted as detailed in the previous section. These included descriptive statistics, reliability analysis, factor analytic diagnostics, correlation analysis, structural path analysis, indirect-effect analysis, moderation analysis, and hierarchical regression. Bootstrapping procedures were used to obtain confidence intervals for the indirect effects, when these effects were examined, due to possible non-normality of the sampling distribution of indirect effects.

Model interpretation was carried out using statistical estimates and pertinent diagnostic data such as distributional characteristics, multicollinearity analysis, and model-fit indices. Each of the analytical procedures was treated as it was intended to be used, and not as a replacement for the other procedures in order to test the same hypothesis.

## 4 Results

### 4.1 Measurement preparation and variable construction

#### 4.1.1 Score coding and direction

A total of 538 questionnaires were obtained and used in the final analytic data set. Each of the key psychological measures was scored on its original 1–5 scale. The positively worded self-control items were reverse scored, yielding higher scores consistently related to increased difficulties or vulnerability: higher scores on the SVA indicated more problematic short-video engagement; higher scores on the BP indicated more frequent intentional delay in intended bedtime; higher scores on the SC indicated poorer self-regulation; higher scores on the ED indicated greater difficulties in executive functioning; and higher scores on the Emotional Distress indicated greater affective symptom burden. The items were grouped into constructs and composite scores were computed as the arithmetic mean of each construct while maintaining the same scale metric, which facilitated direct comparison of the distributions for the five domains.

In the Results section, the observed distributions and statistical associations (captures of these composite scores) are emphasized, rather than the data-cleaning processes involved in their derivation, as described in the Methodology. No clinical criteria were used to categorize the composite measures; thus values are not clinically classified as addiction, executive difficulty or emotional disorder but are interpreted in relation to the sample of the study. This is crucial because the measures were never used as a diagnostic tool for each participant, but rather as continuous research products.

#### 4.1.2 Composite score use in the analytic dataset

Signs reported below are also influenced by the common direction of the composite scores. Positive correlations between SVA and BP, SVA and SC, SVA and ED, and BP and Emotional Distress each showed higher scores in one domain were significantly correlated with higher scores in another domain. The positive SC coefficients especially should not be interpreted as indicating that greater self-control leads to poorer outcomes since the SC composite was reverse keyed with higher scores indicating a greater self-control deficit. This coding clarification is significant as the term ‘self-control’ may be misinterpreted as a higher degree of regulatory capacity when the assessed score on the SC is coded in the opposite direction.

The results on the analytical reporting were organized around the following main questions of the study: What is the distribution and reliability of the five constructs? What are the bivariate associations of the five constructs? What is the relative association between BP and ED in the joint observed-variable path model? How do the separate BP and ED indirect-effect models compare? Demographic comparisons, moderation analyses and hierarchical regression were held as secondary or sensitivity analyses. The main results were limited to confirmatory educational analyses that were sufficiently validated by exploratory computational analyses.

### 4.2 Demographic analysis

The analytic sample was demographically concentrated rather than evenly distributed across sex, academic year, province, and expenditure categories. Of the 538 participants, 362 (67.29%) were female and 176 (32.71%) were male. First-year students accounted for 381 participants (70.82%), followed by 98 sophomores (18.21%), 44 juniors (8.18%), 11 seniors (2.04%), and 4 graduate students (0.74%). The distribution of academics by academic year is therefore dominated by early stage university students, with smaller numbers of senior and graduate students.

Monthly living expenses were similarly concentrated. A total of 425 participants (78.99%) reported monthly expenses of 1,001–2000 RMB, while 83 (15.43%) reported less than 1,000 RMB. The rest of the 30 participants (5.58%) were spread over the above categories of expenditure. The relatively low dispersion is relevant when interpreting the moderation analyses with monthly living expenses as variable since relatively few respondents were in the higher levels of the expenditure scale.

Geographic representation was also uneven. Four hundred and nine participants came from Henan (76.02%), 40 from Zhejiang (7.43%) and 89 from other provinces (16.54%). The sample should thus be considered as being a Chinese university student sample with high Henan representation, not a nationally balanced sample. Demographic composition does not affect the within-sample association estimates, but restricts the generalizability of the sample prevalence of score levels and/or sample differences between subgroups to the total Chinese university population. The main characteristics of the samples are summarized in Table 2 displays the concentration by gender, academic year, expenditure, and province.

**Table 2:** Sample characteristics of the analytic sample (*N* = 538).

Characteristic	Category	*n*	%
Gender	Female	362	67.29
	Male	176	32.71
Academic year	First year	381	70.82
	Sophomore	98	18.21
	Junior	44	8.18
	Senior	11	2.04
	Graduate	4	0.74
Monthly living expenses	<1,000 RMB	83	15.43
	1,001–2000 RMB	425	78.99
	Other categories combined	30	5.58
Province	Henan	409	76.02
	Zhejiang	40	7.43
	Other provinces combined	89	16.54

There are two immediate implications of the concentration of the sample for the rest of the analyses. First, it is important to note that descriptive percentages do not represent national figures of problematic short video consumption or relevant troubles among university students of China. Second, subgroup comparisons have a significantly different precision between categories: estimates for first-year students and Henan participants are based on much larger numbers than estimates for senior, graduate, Zhejiang or higher-expenditure groups. Due to this, statistically significant subgroup findings are reported but are not considered to be part of a redefining central theoretical model.

### 4.3 Descriptive statistics and distributional characteristics of the core constructs

#### 4.3.1 Central tendency and dispersion

Each composite measure had different but similar score distributions that ranged from 1 to 5. Bedtime procrastination had the highest mean score (*M* = 3.044, SD = 0.906, 95% CI [2.967, 3.121]), followed by short-video addiction (*M* = 2.871, SD = 0.795, 95% CI [2.804, 2.938]), executive dysfunction (*M* = 2.737, SD = 0.725, 95% CI [2.676, 2.798]), self-control deficit (*M* = 2.629, SD = 0.552, 95% CI [2.582, 2.675]), and emotional distress (*M* = 2.477, SD = 0.720, 95% CI [2.416, 2.538]). These values are used to describe the location of scores relative to each other and no clinically determined thresholds were applied so should not be interpreted as clinically mild, moderate or severe.

The comparatively higher mean score on BP suggests that sleep-delay behaviors were relatively more endorsed than other difficulties measured in this sample. The distribution of the SVA scores was also clustered around the middle of the scale range, suggesting that there was some variation in problematic engagement, but not a sample that was only at the extremes of the scale. The average scores for ED and emotional-distress were somewhat lower, but the standard deviations suggest that there was a considerable inter-participant variance. The standard deviation of all of the five composite scores was least for SC, indicating a slightly smaller observed distribution than BP, SVA, ED, or emotional distress.

#### 4.3.2 Distributional properties

The distributions of the scores did not suggest extreme floor and ceiling concentration for the major constructs. The histograms in Figure 1 appear to be fairly continuous and have a wide enough range to be appropriate for correlational and multivariable analyses. The distributions are not intended to demonstrate the strict multivariate normality, but rather are used in conjunction with the skewness and kurtosis diagnostics described in the Methodology to support the assumption of approximate continuous measures of the composite scores for the primary parametric analyses. The composite distributions in this section are averages of multiple 5-point items, and as a result, the distribution of the composites has a significantly higher level of gradation than the distribution of the ordinal items.

**Figure 1:**
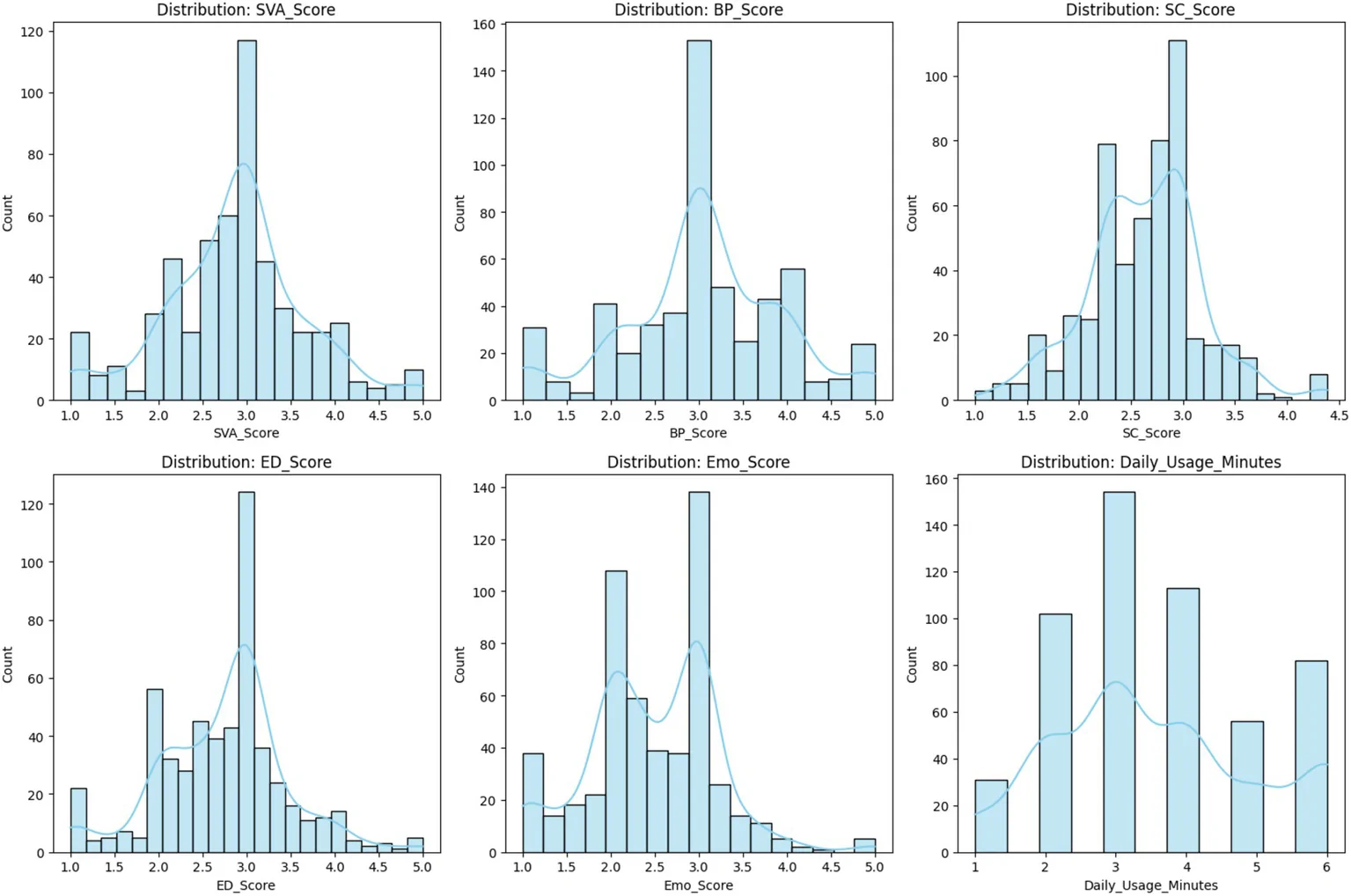
Distribution of the five composite psychological measures. Histograms show the observed distributions of SVA, BP, SC, ED, and Emotional Distress on the 1–5 composite-score metric.

To avoid the repetition of the same constructs in several separate tables, descriptive statistics and reliability coefficients are consolidated in Table 3 and Figure 1. It is also important to see that there are also differences between the descriptive score level and the internal consistency: a construct with a relatively low mean may have an internal consistency that is high, while another construct with a relatively high mean may have an internal consistency that is low. The length of the 95% confidence intervals for the means was relatively short, as there were 538 observations in the analytic sample. The confidence intervals for BP were about 0.15 scale points, SVA was about 0.13, ED was about 0.12, Emotional Distress was about 0.12, and SC was about 0.09. These intervals state ranges of uncertainty on the sample means and NOT clinical reference ranges. Several intervals overlap further support the point that the order of the means should be regarded descriptively and not as support for the notion that one domain is categorically worse than another.

**Table 3:** Descriptive statistics of core psychological constructs.

Variable	Mean	SD	95% Confidence Interval
Short Video Addiction (SVA)	2.871	0.795	[2.804, 2.938]
Bedtime Procrastination (BP)	3.044	0.906	[2.967, 3.121]
Self-Control Deficit (SC)	2.629	0.552	[2.582, 2.675]
Executive Dysfunction (ED)	2.737	0.725	[2.676, 2.798]
Emotional Distress	2.477	0.720	[2.416, 2.538]

All construct scores are mean scores on the original response scale ranging from 1 to 5. A higher score on the SC is a sign of greater self-control deficit. Reliability coefficients do not provide evidence of discriminant validity.

### 4.4 Measurement quality and construct overlap

#### 4.4.1 Internal consistency

The internal consistency of all the multi-item constructs was high. Cronbach’s alpha was 0.929 for SVA, 0.956 for BP, 0.878 for SC, 0.951 for ED, and 0.960 for Emotional Distress. Therefore, all five coefficients were above the typical 0.70 level of group-level research, and four of the five were above 0.90. The results showed that the items in each composite were highly interrelated in this sample. For high alpha scores, however, they are not seen as evidence of the five constructs being mutually distinct but of score consistency.

#### 4.4.2 Factorability and preliminary factor structure

Kaiser–Meyer–Olkin statistic was 0.917 showing good sampling adequacy for the item correlation matrix for factor analytic exploration. The exploratory factor analysis (EFA) scree plot is presented in Figure 2. A strong first factor was followed by a significant drop and a subsequent gradual decline in the eigenvalues. The plot does show some common variance across the survey items, but does not by itself support a single-factor or a clear five factor solution. The factor-analyses results have been interpreted as preliminary diagnostics of the constructs instead of definitive evidence for distinct latent dimensions, due to the theoretical expectation that they correlate.

**Figure 2:**
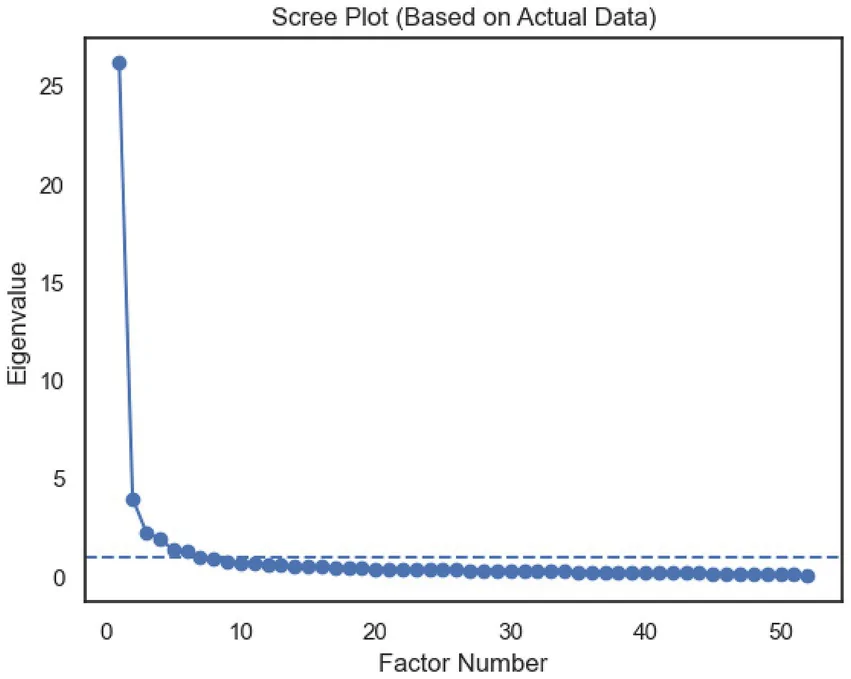
Scree plot from the exploratory factor analysis. The plot is interpreted as a preliminary diagnostic of the item covariance structure and not as definitive evidence of discriminant validity.

The Harman single factor diagnostic revealed that the first factor (unrotated) explained 49.74% of the total variance. This value is very close to the widely recommended 50% heuristic and should not be taken as indicative of the absence of common-method variance. Rather, the findings suggest that there is a high degree of shared variance between items, which makes sense in this one-shot study with a self-report measure. The structural and correlational data are thus viewed with the knowledge that some data shared by the measurement method may overestimate some of the associations observed.

#### 4.4.3 Common-method and discriminant-validity considerations

SC0010 and ED have an especially important issue of discriminant validity. The largest off-diagonal correlation in the matrix was their bivariate correlation, r = 0.810. This high correlation is consistent with the notion that there is conceptual overlap between the self-regulatory capacity and executive functioning constructs and suggests that they may assess overlapping psychological constructs. Neither reliability coefficients nor the KMO statistic can address this issue, and neither can variance inflation factors be used to demonstrate discriminant validity because VIFs are used to control for the instability of the coefficients in multivariable models, not to measure construct distinctiveness.

Therefore, the present findings should not be interpreted as supporting the empirical separation of the constructs SC and ED. They are not analyzed as continuous scores but rather as theoretically differentiated observed composite scores due to their different roles in the analyses mentioned above, and the results of analyses involving both variables are interpreted with caution. This treatment directly limits overstatement: strong associations involving ED are not presented as proof of an isolated executive mechanism independent of all self-regulatory content, and the SC–ED overlap is carried forward as an explicit interpretive limitation.

The measurement results are thus used to support a cautious conclusion. The internal consistency of the five scales is good, and the results of the factorability analysis of the item set are satisfactory; the measurement structure is not fully validated and the constructs cannot be considered as definitely independent. Specifically, the high first factor shares observed (49.74%) and the high correlation between SC and ED (0.810) suggests that caution should be taken when making any claims that the observed associations are due to completely independent psychological processes (Figure 2). When interpreting relatively large ED coefficients reported in the structural and regression analyses, this measure qualification is important.

### 4.5 Bivariate associations and multicollinearity

#### 4.5.1 Correlation structure of the main constructs

In the five composite correlations, only one of which was negative, all were statistically significant at *p* < 0.001 and positively correlated. There was a strong correlation between SVA and BP (*r* = 0.696), with those reporting higher levels of problematic short-video engagement also reporting higher levels of BP. SVA was also positively correlated with SC (*r* = 0.619), ED (*r* = 0.616), and Emotional Distress (*r* = 0.564). The cross-sectional design does not allow for conclusions to be drawn regarding the direction of these relationships, and these coefficients indicate that problematic engagement occurred with difficulty in self-regulation, executive complaints and emotional symptoms.

The BP was correlated with SC (*r* = 0.631), ED (*r* = 0.668), and Emotional Distress (*r* = 0.564). The BP–ED association is remarkable, since the two domains of sleep delay behavior and executive difficulties are not independent in the sample. When ED is added to the BP-distress relationship in the subsequent joint path model, the relationship between BP and distress is attenuated if there is significant variance shared between the two.

ED was bivariate significantly associated with Emotional Distress (*r* = 0.773) and SC was significantly associated with Emotional Distress even though it was less strong (*r* = 0.722). The correlation between the SC and ED was *r* = 0.810. Overall, the matrix reveals a high level of positive covariation among the five constructs, rather than a collection of independent predictors. This is why the correlation matrix can be described as a map of association, rather than a map of mediation, causation or temporal order.

The correlation matrix is shown in Figure 3 and the actual values of the correlation coefficients in Table 4. The heat map is left because the relative size of the associations is easily apparent, especially that of SC–ED and that of ED–distress. The visual pattern “does not prove” the theoretical pattern, however; it simply conveys the summary of the observed bivariate covariance structure.

**Figure 3:**
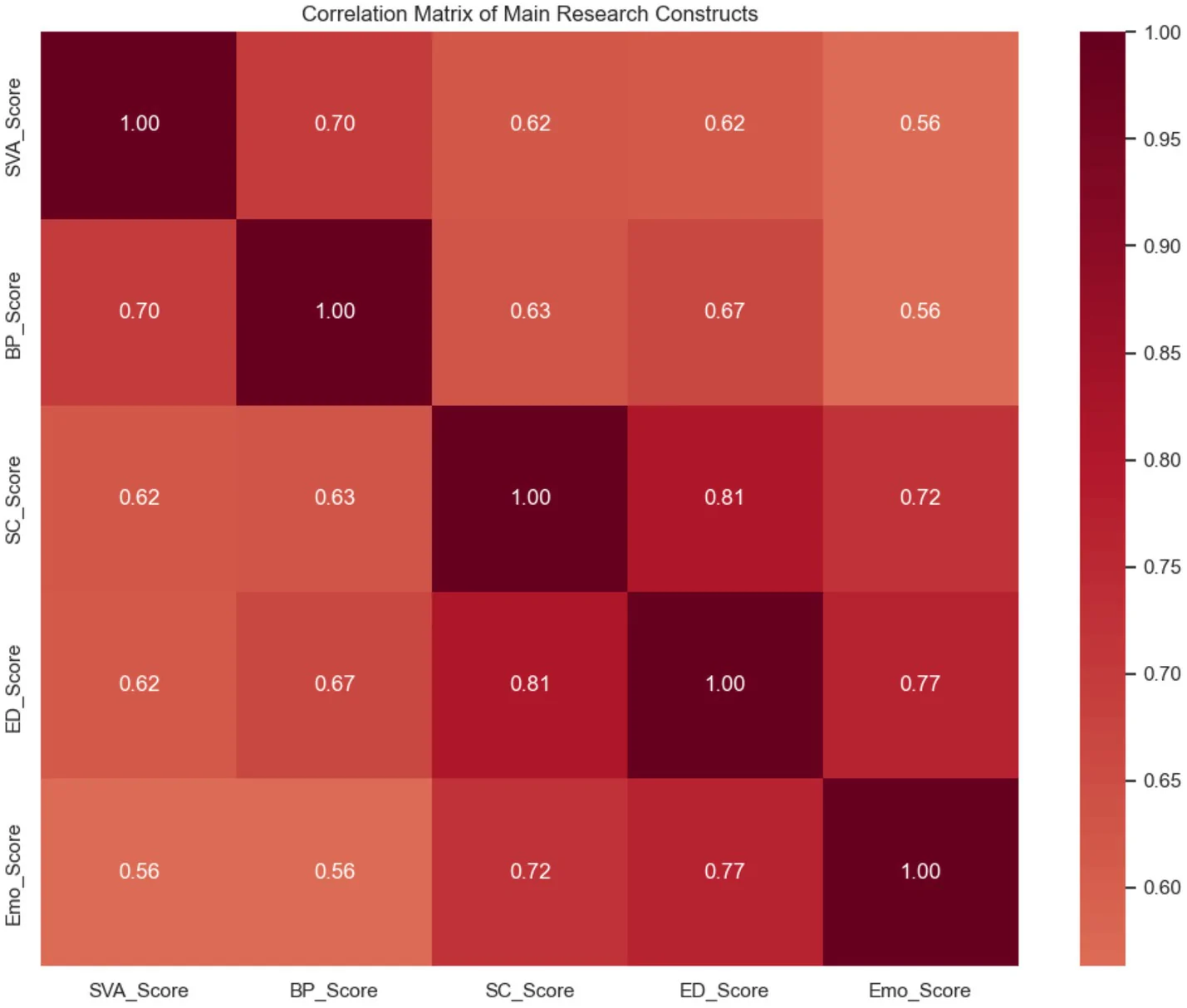
Pearson correlation matrix of the main composite constructs. Darker cells represent stronger positive correlations; the strongest off-diagonal association is between SC and ED (*r* = 0.810).

**Table 4:** Pearson correlation matrix.

Variable	SVA	BP	SC	ED	Emo
SVA	1.000	0.696	0.619	0.616	0.564
BP	0.696	1.000	0.631	0.668	0.564
SC	0.619	0.631	1.000	0.810	0.722
ED	0.616	0.668	0.810	1.000	0.773
Emo	0.564	0.564	0.722	0.773	1.000

All reported off diagonal correlations were significant at the *p* < 0.001 level. The reported VIF values for the predictors in the multivariable models were <3.5; VIF is not a test of discriminant validity but rather a test of coefficient stability (Figure 3).

Multivariable models were reported to have variance inflation factors < 3.5 for the predictors included in the model. This indicates that severe numerical multicollinearity probably did not cause the regression coefficients to be very unreliable. The VIF interpretation is however, very limited. The low or moderate VIF does not support the discriminant validity of the factors and does not rule out the substantial overlap suggested by the *r* = 0.810 for SC and ED. Both are thus reported together: The regression models were not estimable due to extreme collinearity, but the psychological difference between SC and ED is not empirically perfect.

#### 4.5.2 Multicollinearity assessment

All off-diagonal correlations reported were significant at the *p* < 0.001 level. VIF values were reported for predictors in the multivariable models, and were <3.5; VIF is not a test of discriminant validity but rather is a coefficient-stability diagnostic (Table 3).

### 4.6 Demographic and academic-year differences

Gender and academic year were used as secondary subgroup analysis variables in order to investigate whether the scores of the SVA differed across these subgroups. Because the study was not designed primarily to test the differences between the subgroups and the sizes of the subgroups were unequal, these analyses were regarded as descriptive and exploratory. The results are therefore interpreted as in-sample variation, and not as consistent population-level differences.

#### 4.6.1 Gender differences in short-video addiction

To investigate gender differences in problematic short-video engagement in the current sample, the SVA scores of men were compared with those of women. Figure 4 shows the distribution of scores on the SVA by gender. The sample was overwhelmingly female so the comparison results should be treated with caution and as a preliminary measure of a generalizable gender difference, and should not be used as proof of any gender difference without replication in a more balanced sample.

**Figure 4:**
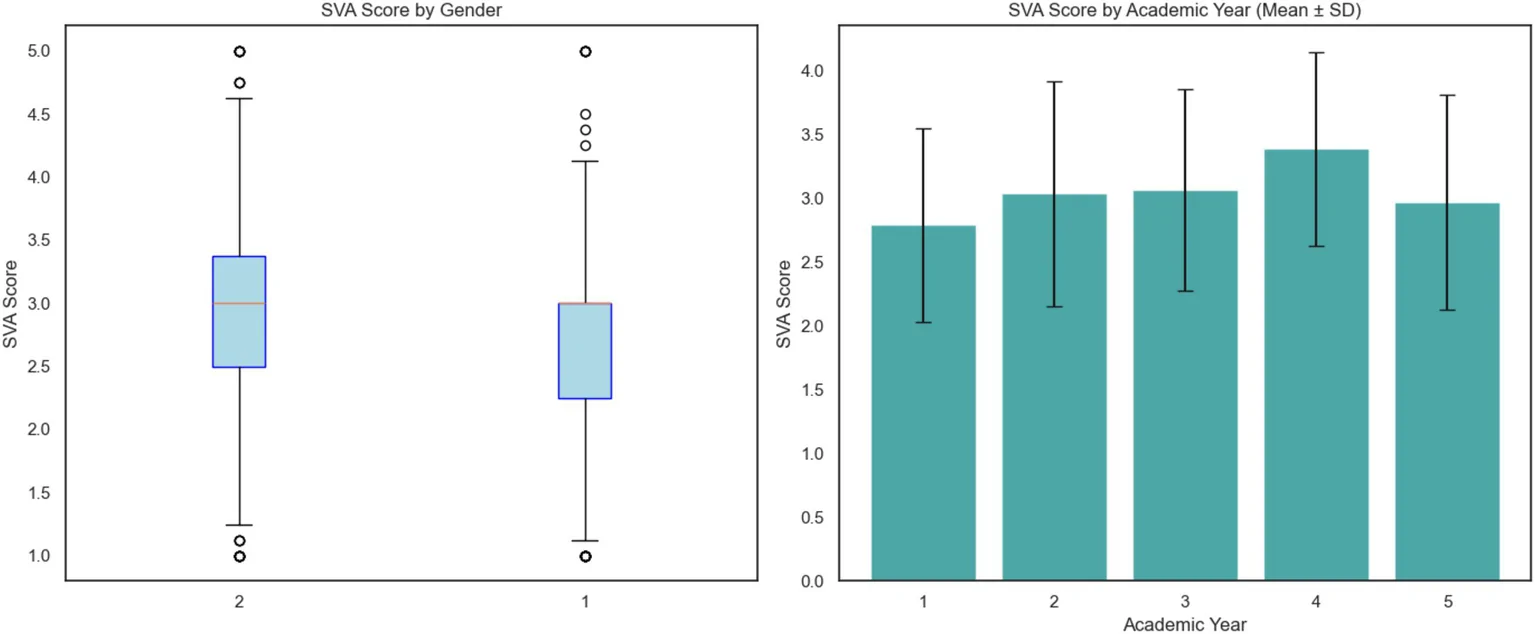
Distribution of short-video addiction scores by gender. Box plots provide the median, interquartile range, overall range and outliers for the SVA scores by gender.

#### 4.6.2 Academic-year differences in short-video addiction

Results also differed between academic-year groups in terms of the scores on the Short-Video Addiction scale. The omnibus analysis revealed a significant difference in the mean SVA scores for the five academic-year categories, *F*(4, 533) = 3.95, *p* = 0.0036. The result indicates that there were at least two academic-year groups with different mean SVA scores.

The academic-year groups were, however, very unevenly sized, with the first year group being almost entirely made up of first years, and not many students in the senior or graduate categories. Because of this, estimates for the smaller groups are less accurate and the omnibus result should not be interpreted as indicating a specific period of academic risk for any academic year. There is a graph of SVA scores by academic year group which is shown in Figure 5. It is provided mainly for the purpose of demonstrating the extent of overlap and variation within the group and not to suggest distinctly different academic year profiles. A Tukey *post hoc* comparison was reported in the original analysis, but the directionality of the reported freshman–sophomore difference should be checked against the original group coding and output for a specific pairwise interpretation to be included.

**Figure 5:**
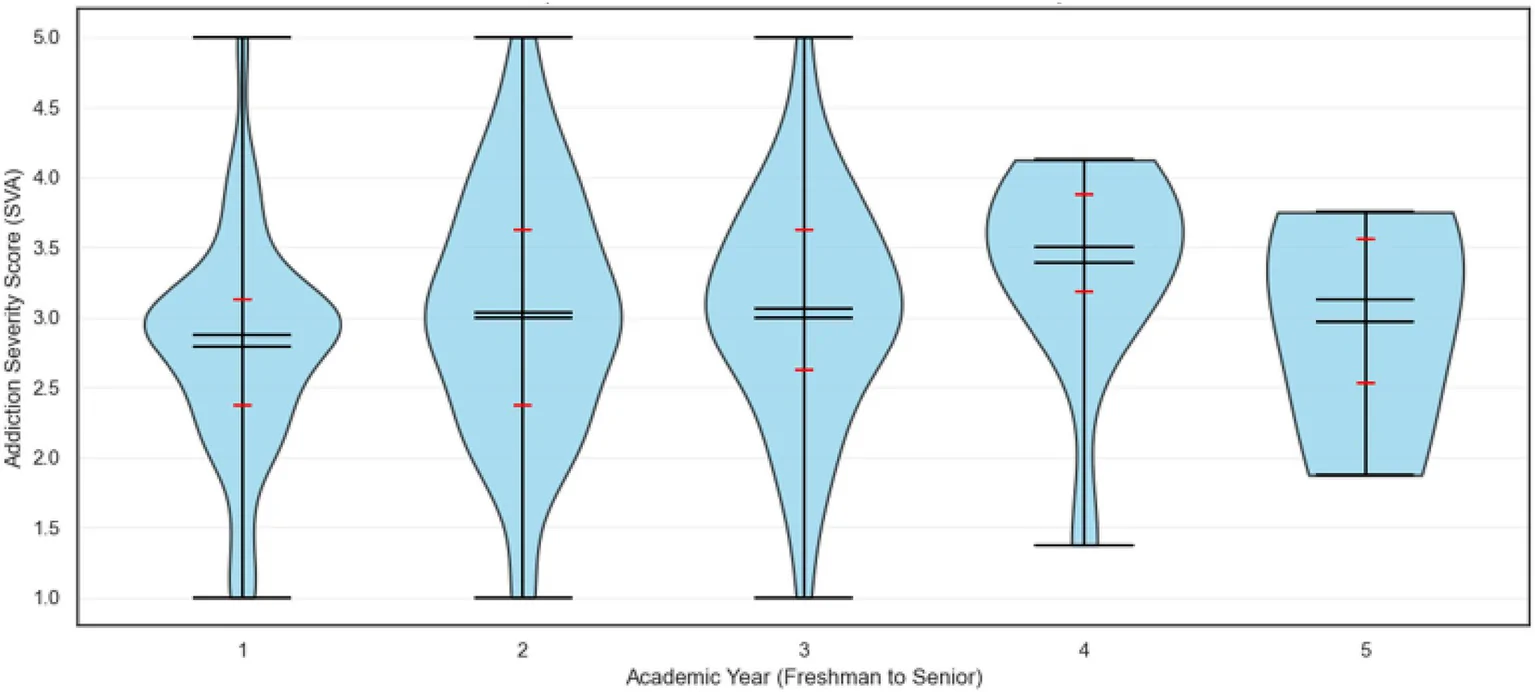
Distribution of short-video addiction scores across academic-year groups. The violin plots give an indication of the distribution of the SVA scores in each academic-year group, and the internal markers show the central tendency and spread. Caution should be used when interpreting the small sample size of the senior and graduate groups.

### 4.7 Primary observed-variable path model

#### 4.7.1 Model fit

The major analysis of the present study was an observed-variable path model in the SEM framework using standardized composite scores. There was a mixed fit with the model. The Comparative Fit Index was 0.968 and the Tucker–Lewis Index was 0.941, which indicated good incremental fit when compared to a null model. The Root Mean Square Error of Approximation, on the other hand, was of 0.125. This value of the RMSEA is not warranted as acceptable due to the complexity of the model, but rather represents evidence of an unacceptable absolute fit.

Both the fitting indices differ; caution required. The high CFI and TLI show significant improvement on a model which assumes no co-variation between the observed variables, while the high RMSEA shows that the structure of the co-variation is not well reproduced for the model to be considered globally well fitting. For that reason, the individual path coefficients are reported as informative conditional associations within the specified model, not as definitive confirmation of a causal or complete theoretical mechanism.

#### 4.7.2 Structural path estimates

In the equation for BP, SVA was positively standardized (*β* = 0.495, *p* < 0.001) for bedtime procrastination. SC also had a positive association with BP (*β* = 0.324, *p* = 0.001). The sign of this coefficient shows that higher self-control scores were related to higher bedtime procrastination scores and therefore higher self-control deficit was related to higher bedtime procrastination scores when SVA was entered into the equation. In the stated model, the standardized association between SVA and BP was larger than that of the other coefficients, suggesting that the relative size of these two coefficients is indicative of the relative magnitude of the association between SVA and BP.

The standardized association (β) of Emotional Distress was the highest (*p* < 0.001) for the equation. SVA had a smaller positive direct association (*β* = 0.133, *p* = 0.001) with Emotional Distress. However, when ED and SVA were both included in the same equation, BP was not independent after (*β* = 0.018, *p* > 0.05). This disparity between the bivariate and the multivariable results is a key outcome. The bivariate BP–distress association was slightly different, with *r* = 0.564, but was almost completely accounted for by the variance explained by the other predictors, particularly ED, when the conditional path was calculated.

An insignificant BP coefficient does not mean bedtime procrastination does not matter for students’ well-being. It maintained a strong connection with ED, SC, Emotional Distress and SVA at bivariate level. Instead, the joint model suggests that under this specification, there is no unique variation in Emotional Distress data beyond ED and SVA. In contrast, the large size of the ED coefficient does not imply the causal direction of the relationship (i.e., does not indicate that executive dysfunction is a consequence of problematic short video usage or that it occurs as a mediator in the relationship). The model fit was not perfect and ED and distress were assessed concurrently via self-report.

The structural coefficients taken together can answer the central comparative question without a serial-chain interpretation. The ED coefficient is about 5 times the size of the direct SVA coefficient, and is much larger than the BP coefficient, when ED is the outcome. When the outcome is ED, SVA is still associated with BP, but the ED coefficient is approximately 5 times larger than the direct SVA coefficient and larger than the BP coefficient. This comparison is a descriptive comparison and not a formal test to compare whether the ED–distress relationship is statistically stronger than the BP–distress path, but it illustrates why the ED–distress relationship merits more focus in the Results while the BP–distress path requires an unsupported description in the joint specification.

Table 5 also shows the reasons for reporting fit statistics and path significance separately. Despite the poor absolute fit values determined by RMSEA, several individual paths are statistically accurate. Significant Coefficients are then not allowed to invalidate the model misfit evidence. The correct reading is that some conditional associations are not very well represented in the model specified, but the general covariance structure is better represented than would be the case if it were a uniquely correct representation of the relationships among the five constructs.

**Table 5:** Consolidated primary, indirect-effect, moderation, and sensitivity results.

Analysis	Parameter	Estimate	Statistical evidence	Interpretation
Panel A: Primary path model	CFI	0.968	–	Favorable incremental fit
Panel A: Primary path model	TLI	0.941	–	Favorable incremental fit
Panel A: Primary path model	RMSEA	0.125	–	Poor absolute fit
Panel A: Primary path model	SVA → BP	*β* = 0.495	*p* < 0.001	Positive conditional association
Panel A: Primary path model	SC deficit → BP	*β* = 0.324	*p* = 0.001	Positive conditional association
Panel A: Primary path model	ED → Distress	*β* = 0.679	*p* < 0.001	Largest standardized association with distress
Panel A: Primary path model	SVA → Distress	*β* = 0.133	*p* = 0.001	Small positive direct association
Panel A: Primary path model	BP → Distress	*β* = 0.018	*p* > 0.05	Not independently supported
Panel B: Separate indirect effects	SVA → BP → Distress	Indirect = 0.232; direct = 0.331	Reported as statistically significant	Cross-sectional indirect association
Panel B: Separate indirect effects	SVA → ED → Distress	Indirect = 0.423; direct = 0.141	Reported indirect estimate	Larger indirect estimate than BP model
Panel C: Moderation	SVA × monthly expenses	–	*p* = 0.433	No evidence of moderation
Panel C: Moderation	SVA main effect in thrill-seeking model	*β* = 0.385; SE = 0.062	*t* = 6.21, *p* < 0.001	Positive association with ED
Panel C: Moderation	Thrill-seeking main effect	*β* = 0.124; SE = 0.058	*t* = 2.14, *p* = 0.033	Positive association with ED
Panel C: Moderation	SVA × thrill seeking	*β* = 0.156; SE = 0.078	*t* = 2.01, *p* = 0.0459	Tentative exploratory interaction
Panel D: Hierarchical regression	Model 1	*R*^2^ = 0.005; adj. *R*^2^ = 0.003	–	Demographic block explains little variance
Panel D: Hierarchical regression	Model 2 (+ SVA)	*R*^2^ = 0.323; adj. *R*^2^ = 0.319; Δ*R*^2^ = 0.318	*p* < 0.001 for Δ*R*^2^	Substantial increase in explained variance
Panel D: Hierarchical regression	Model 3 (+ ED, SC, BP)	*R*^2^ = 0.629; adj. *R*^2^ = 0.625; Δ*R*^2^ = 0.306	*p* < 0.001 for Δ*R*^2^	Further increase in explained variance
Panel D: Hierarchical regression	ED in Model 3	*β* = 0.514	*t* = 8.42, *p* < 0.001	Strongest reported standardized coefficient
Panel D: Hierarchical regression	SC in Model 3	*β* = 0.320	*t* = 5.16, *p* < 0.001	Independent positive association
Panel D: Hierarchical regression	BP in Model 3	*β* = 0.041	*t* = 0.88, *p* > 0.05	Not independently supported
Panel D: Hierarchical regression	SVA in Model 3	–	*t* = 2.11, *p* < 0.05	Significant after adjustment

Indirect-effect estimates are reported from separate single-mediator models and should not be interpreted as temporal or causal mediation. Secondary moderation and hierarchical-regression results are reported as supplementary analyses and should be interpreted alongside the primary observed-variable path model.

### 4.8 Separate indirect-effect analyses

#### 4.8.1 Bedtime-procrastination indirect-effect model

Indirect effect models were tested separately for BP and ED, rather than assuming a serial order for the two constructs. The reported indirect estimate of BP to Emotional Distress in the BP model was 0.232, and the other direct estimate of SVA to Emotional Distress was 0.331. The single-mediator analysis reported as a significant mediator analysis was used to describe both as significant and thus partial mediation. Because of the cross-sectional design, these numerical estimates are interpreted here as indirect statistical associations, and not as evidence that the SVA is a temporal cause of procrastination at bedtime, which is a cause of emotional distress.

#### 4.8.2 Executive-dysfunction indirect-effect model

For the same ED model, the indirect estimate from SVA to Emotional Distress via ED was reported as 0.423 while the direct estimate of Emotional Distress was 0.141. Therefore, the ED indirect estimate in the separate model was greater than the BP indirect estimate (0.423 vs. 0.232). This comparison is descriptive because there is no formal statistical test reported to examine the difference between the two indirect estimates. However the direction of the comparison was congruent with the joint path model in which ED had a strong conditional relationship with distress while BP did not.

#### 4.8.3 Comparison of the separate indirect-effect models

When the specification of the separate BP result is distinguished from the specification of the joint model, they are not contradictory. The BP-related variance that is common across both models is attributed to the BP indirect path, since ED is not included in this single-mediator BP model. If ED is specified as a concurrent process in the joint model, BP is no longer found to have a statistically supported independent process to distress. The evidence thus does not warrant a chronological causal inference of the production of ED by SVA, followed by the production of BP, or vice-versa. The analyses suggest otherwise, however, as both BP and ED are overlapping correlates of problematic short video use and emotional distress, and ED explains significantly more unique variance in emotional distress when both are included in the joint specification.

The direct and indirect components of the separate BP model and the BP and ED indirect estimates are given side by side in Table 5. It is included for clarity, but the caption and text make it clear that the results from the single mediator are different from the results from the joint path model, so that the non-significant BP path in the latter is not hidden.

The total SVA–distress estimate also is very similar across the two separate models – 0.232 + 0.331 = 0.563 for the BP model and 0.423 + 0.141 = 0.564 for the ED model. These totals are comparable as both the models are derived from the same general association, SVA–distress, and the difference between the two lies in the partitioning of this association into direct and indirect components (Figure 6). It is important to note, however, that the allocation of the association is not the same in the two models; the ED model allocates a higher percentage of association to the indirect component than the BP model.

**Figure 6:**
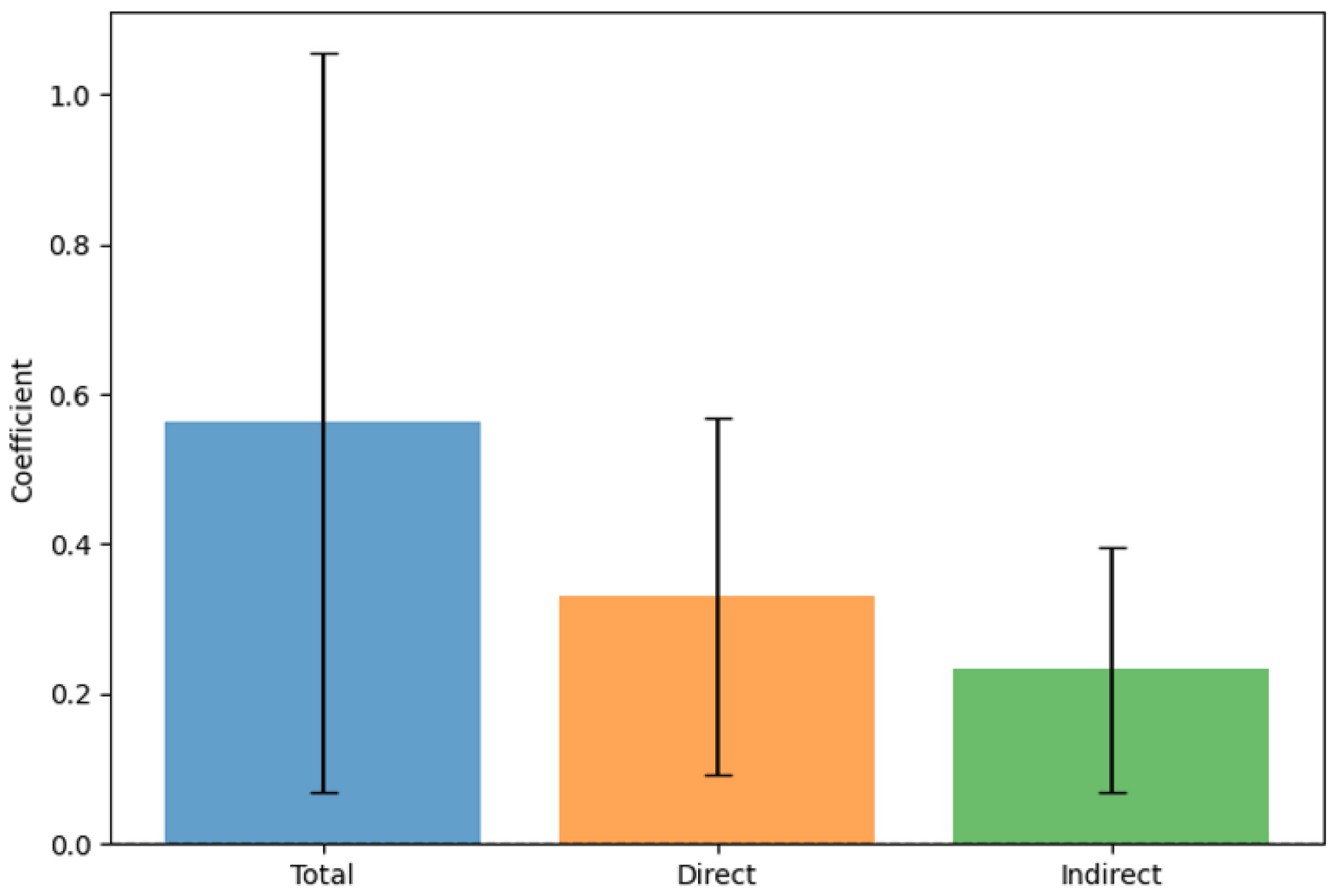
Primary structural and indirect-effect results.

It is therefore only feasible to compare the two indirect-effect models on the basis of the reported point estimates and their general shape by specification. The larger ED indirect estimate should not be taken as being statistically larger than the BP estimate as there is no formal test reported comparing the two indirect effects. Rather, the comparison is descriptive, and is analyzed in conjunction with the joint path model, in which ED continued to have a strong association with distress, but BP did not maintain an independent association.

### 4.9 Exploratory moderation analyses

Secondary moderation analyses were also conducted to explore whether certain individual and contextual factors were related to the modification of certain key relationships related to Short-Video Addiction (SVA). The analyses were exploratory and were not included in the primary structural model. Interactions were therefore interpreted with caution and focus was given to the size, direction, and statistical uncertainty of the estimated interactions and not on causal effect modification.

#### 4.9.1 Thrill-seeking as a moderator of the association between SVA and executive dysfunction

The first exploratory moderation analysis was conducted to see if the association between the Short-Video Addiction and Executive Dysfunction was moderated by thrill-seeking tendency. The interaction term of SVA by thrill-seeking was positive and significant *β* = 0.156, SE = 0.078, *t* = 2.01, *p* = 0.0459. This interaction suggests that the positive association between SVA and Executive Dysfunction differed according to participants’ level of thrill seeking.

The main association between SVA and Executive Dysfunction was also positive and statistically significant, *β* = 0.385, SE = 0.062, *p* < 0.001. Thrill seeking had a smaller positive correlation with Executive Dysfunction, *β* = 0.124, SE = 0.058, *p* = 0.033. Combined, these estimates showed that the relationship between SVA scores and executive impairments was positive and that this relationship was slightly more pronounced for those individuals with higher thrill-seeking scores.

The Executive Dysfunction increased as SVA rose in both the lower and higher thrill-seeking group as illustrated in Figure 7. The higher thrill-seeking group had a slightly steeper positive pattern in the fitted regression line, which was in line with the positive interaction coefficient. There was still significant overlap, though, between the observations of the two groups.

**Figure 7:**
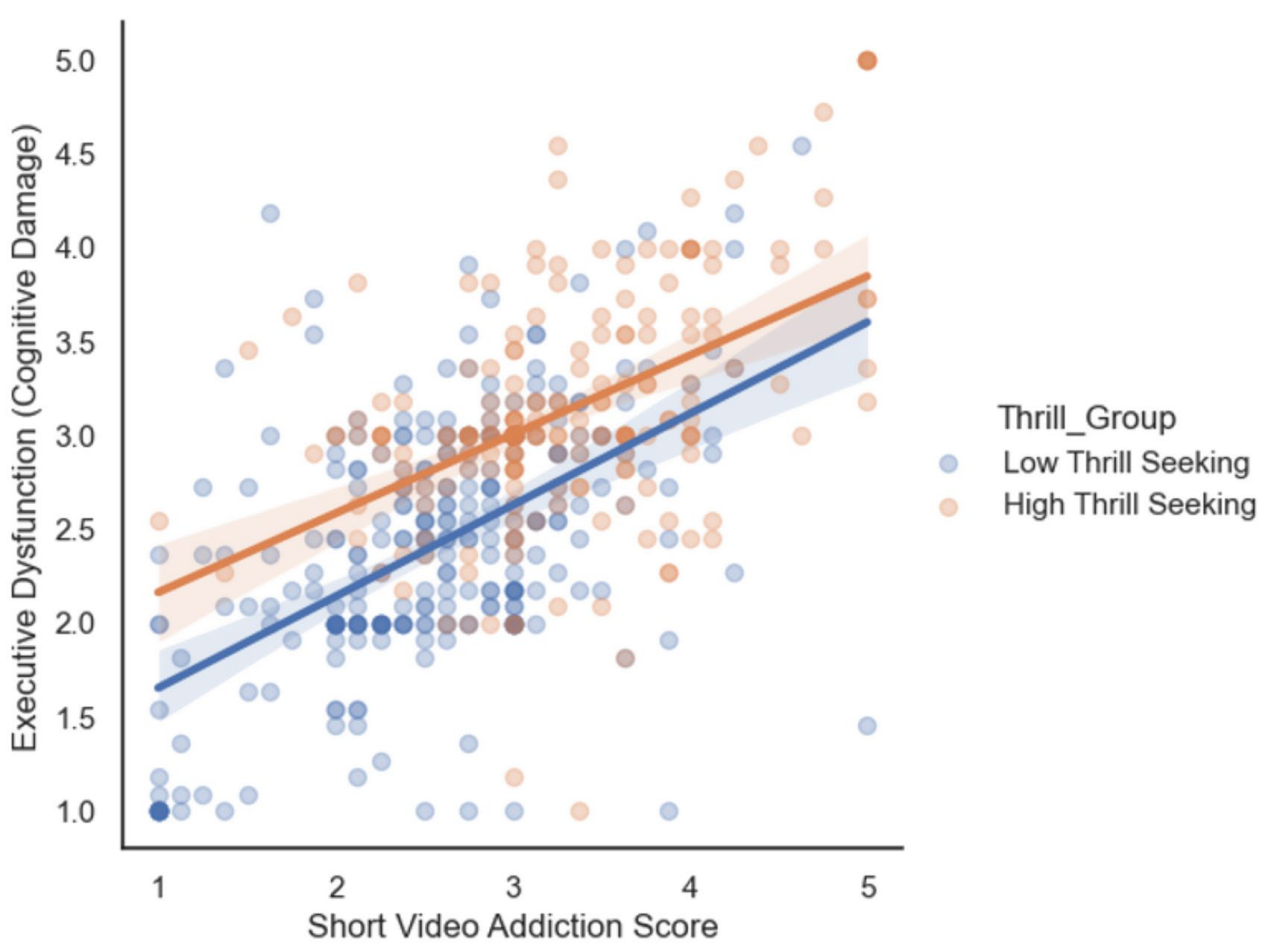
Exploratory moderation of the association between short-video addiction and executive dysfunction by thrill-seeking level. Points estimates observed values of Short-Video Addiction and Executive Dysfunction. The estimated associations are depicted by fitted regression lines, separately for the lower and the higher group of participants regarding thrill-seeking behavior. The interaction was positive (*β* = 0.156, SE = 0.078, *t* = 2.01, *p* = 0.0459) and should be interpreted as exploratory.

The finding is small and the *p*-value is near the conventional 0.05 level, so should be considered preliminary. Furthermore, the cross-sectional design does not allow to determine whether thrill seeking leads to greater vulnerability to executive problems related to problematic short-video use, whether vice versa executive problems influence thrill seeking, or whether the observed pattern is due to other variables not measured. The moderation result thus serves as exploratory evidence of the heterogeneity in the association between SVA and Executive Dysfunction, but not as support for a specific psychological mechanism.

#### 4.9.2 Monthly living expenses as a moderator of the association between SVA and emotional distress

One of the second moderation analyses investigated whether the link between Short-Video Addiction and Emotional Distress was moderated by monthly living expenses. The relationship between SVA and monthly living expenses was not significant statistically (*p* = 0.433). Therefore, the association between SVA and Emotional Distress did not vary across monthly spending means in this sample.

This insignificant interaction is crucial because it suggests that there was no significant increase or decrease in the observed relationship between problematic short-video engagement and Emotional Distress depending on how much participants reported spending on a monthly basis. Therefore, monthly living cost was not considered as an important moderator of the association between SVA and Emotional Distress.

This finding should not be construed as evidence of the lack of association between socioeconomic factors and problematic use of short videos or emotional wellbeing in general. Monthly living costs is one relatively general measure of students’ economic situation and may not fully reflect students’ socioeconomic status, financial hardship, economic surpluses or economic insecurity. Additionally, the small number of people in each expenditure category may have resulted in less precision in the detection of relatively small interaction effects.

There is no statistically significant interaction and this is an exploratory analysis, so there is not any specific interpretation for each of the subgroup of monthly living expenses with regard to the association between the SVA and Emotional Distress. Therefore the result is reported as a negative exploratory result and will not influence the interpretation of the main structural analyses.

### 4.10 Hierarchical regression sensitivity analysis

To check the incremental contribution of Short-Video Addiction (SVA) and the cognitive-regulatory variables, a hierarchical multiple regression was conducted. The three sequential models included variables. Model 1 was age and gender, model 2 was age, gender and SVA, model 3 was age, gender and SVA and additionally Executive Dysfunction (ED), Self-Control Deficit (SC) and Bedtime Procrastination (BP). The objective of this analysis was to see whether the relative pattern found in the structural model was also present in the analysis of Emotional Distress in a traditional multivariable regression analysis.

#### 4.10.1 Incremental explained variance

The $R^{2}$ for age and $R^{2}$ for gender were very small in Model 1 (0.005 and 0.003, respectively). The total variance in Emotional Distress for the demographic variables in the model was less than 1%.

The SVA was added to Model 2, which greatly enhanced the model’s explanatory power. The increased from 0.005 to 0.323, with an adjusted $R^{2}$ of 0.319. This was an incremental increase of Δ $R^{2}$ =0.318 (*p* < 0.001). For Model 2, SVA was found to have a strong positive correlation with Emotional Distress (*β* = 0.568), suggesting that higher engagement with short videos was correlated with higher emotional distress, even after the demographic block was taken into account.

Demographic variables and SVA were combined with ED, SC and BP in the model used for Model 3. The full model explained 62.9% of the variance in Emotional Distress ( $R^{2}$ =0.629; adjusted $R^{2}$ =0.625). When the cognitive-regulatory variables were added to Model 2, they added another Δ $R^{2}$ =0.306, *p* < 0.001. This increase in magnitude suggests that the ED, SC and BP provide significant extra explanation, in addition to the demographics and SVA.

#### 4.10.2 Associations in the full regression model

In the complete model, the highest standardized association with Emotional Distress was observed for Executive Dysfunction (*β* = 0.514, *t* = 8.42, *p* < 0.001). Emotional Distress was also independently positively associated with Self-Control Deficit (*β* = 0.320, *t* = 5.16, *p* < 0.001). However, Bedtime Procrastination’s coefficient was small and non-significant when the other variables were added (*β* = 0.041, *t* = 0.88, *p* > 0.05).

Even though the effect of SVA was reduced after the addition of ED, SC and BP, it was still statistically significant in the full model (*t* = 2.11, *p* < 0.05). This attenuation suggests that problematic engagement with short videos was strongly correlated with the cognitive-regulatory factors in the final model. Simultaneous adjustment for these variables, however, did not eliminate SVA (as indicated by the persistence of a statistically significant SVA coefficient), meaning that SVA persisted in providing information about Emotional Distress.

Demographics were not significant in the final model (reported *β* = 0.020, *t* = 0.45), consistent with what was demonstrated in the model 1 results, where the demographics variables accounted for a very small amount of variance. The coefficient pattern overall changed significantly following the cognitive-regulatory variables were added: ED and SC had the strongest separate effects on Emotional Distress, while the effect for BP was no longer statistically significant.

#### 4.10.3 Consistency with the primary path model

The pattern of the hierarchical regression was similar to the main structure path analysis. Executive Dysfunction remained an independent association with Emotional Distress in both approaches and Bedtime Procrastination did not have an independent association when the correlated cognitive-regulatory variables were considered concurrently. It is important because both analyses provide a different statistical perspective on the data and each provides information about the model fit in different ways: A path model evaluates a specified system of simultaneous associations, yielding global model-fit information; Hierarchical regression focuses on the incremental variance explained by the addition of blocks of variables.

Despite the large amount of explained variance, the relationships among the cognitive-regulatory variables should be considered when interpreting the results of Model 3. More specifically, there was a high degree of empirical overlap between SC and ED (*r* = 0.810). The regression coefficients are therefore the unique statistical relationships of each construct once the effects of the other constructs have been statistically removed and should not be interpreted as indicating that these constructs are fully independent psychological mechanisms.

The overall $R^{2}$ of 0.629 signifies that this sample had a high percentage of variance explained by all of the variables. But, since all variables were measured at the same time and self-reported, the percentage variance explained does not indicate causal direction or temporal ordering. The hierarchical regression is thus not intended to provide independent evidence of a causal pathway, but rather as a sensitivity analysis to support the relative pattern of associations found in the primary model.

### 4.11 Summary of the main findings

Descriptive, bivariate, structural, indirect-effect, moderation, and regression analyses yielded consistent results for the Results. Each of the principal psychological constructs studied were correlated with a positive relationship with Short-Video Addiction. The bivariate level findings showed that SVA was highly correlated with Bedtime Procrastination (*r* = 0.696), and was also positively correlated with Self-Control Deficit (*r* = 0.619), Executive Dysfunction (*r* = 0.616), and Emotional Distress (*r* = 0.564). All of these correlations were statistically significant at *p* < 0.001. The highest correlation among the study constructs was for the relationship between SC and ED (*r* = 0.810) with the bivariate relationship between ED and Emotional Distress (*r* = 0.773). These relationships were further clarified in the primary path model, in which the constructs were related simultaneously. SVA was positively correlated with Bedtime Procrastination (*β* = 0.495, *p* < 0.001) and Self-Control Deficit was also positively correlated with BP (*β* = 0.324, *p* = 0.001). Among the independent associations, Executive Dysfunction had the largest direct relation with Emotional Distress (*β* = 0.679, *p* < 0.001), and the smaller direct relation with SVA (*β* = 0.133, *p* = 0.001). The direct role of BP and ED was even smaller and insignificant when ED and SVA were modeled simultaneously (*β* = 0.018, *p* > 0.05). The structural model got mixed support from the global fit indices. CFI (0.968) and TLI (0.941) showed good incremental fit, while the RMSEA (0.125) showed poor absolute fit. Therefore, the structural coefficients must be viewed in light of this limitation in the model fit and not as an indication that the proposed specification is complete for the relationships between the constructs.

The separate indirect-effect analyses also indicated different patterns for BP and ED. In both cases, the estimated indirect association through ED was larger than the indirect association through BP, and was indirect when the two variables were examined separately. Important, these single-mediated models should not be seen as defining a time sequence. When ED was added, the joint structural model indicated that BP did not have a separate relationship, but ED did. This is because the result is more aligned with ED and BP being overlapping but differently weighted correlates of engagement and emotional well-being with short videos rather than the one chronological pathway.

Limited evidence of heterogeneity was found through secondary analyses. There were also differences in SVA between the academic-year groups in the academic-year omnibus analysis, *F*(4, 533) = 3.95, *p* = 0.0036, but these could not be interpreted in detail because of differences in the number of subjects per group. The SVA–Emotional Distress relationship was not significantly altered when model comparisons were conducted that included monthly living expenses (*p* = 0.433) in the moderation analyses. The interaction between SVA by thrill-seeking was positive and statistically significant (*β* = 0.156, SE = 0.078, *t* = 2.01, *p* = 0.0459), but the magnitude and near-significance of the interaction call this finding into question and makes it advisable to treat the finding as exploratory rather than conclusive.

Finally, the pattern of relative sensitivity obtained from the hierarchical regression analysis confirmed the primary pattern. The model explained 62.9% of the variance after the addition of ED, SC and BP, and SVA added significantly after demographics to explain 62.9% of variance in Emotional Distress. Within the full model, ED (*β* = 0.514, *p* < 0.001) and SC (*β* = 0.320, *p* < 0.001) retained independent associations with Emotional Distress, whereas BP did not (*β* = 0.041, *p* > 0.05).

## 5 Discussion

### 5.1 Summary of principal findings

This study investigated the correlation among the short-video addiction (SVA), self-control deficit, bedtime procrastination, executive dysfunction, and emotional distress of 538 Chinese college students. In general, there was a relationship between the higher problematic short-video engagement and the lower scores for self-regulation, more bedtime procrastination, more executive difficulties, and more emotional distress. Perhaps most striking was the finding that the relative importance of executive dysfunction and bedtime procrastination was much the same when both were taken into account. The direct association between SVA and emotional distress was substantially smaller (*β* = 0.133, *p* = 0.001) and the direct association between bedtime procrastination and emotional distress was no longer statistically significant (*β* = 0.018, *p* > 0.05) in the joint structural model. The results of the hierarchical regression analysis revealed a similar trend overall, supporting the idea that executive-regulatory challenges might be more specific as to emotional vulnerability in students with problematic short-video engagement. However, these findings must be interpreted as associations and not necessarily as a causal ordering, given the cross-sectional design of the study and the mixed structural model fit (a higher RMSEA).

### 5.2 Executive dysfunction and emotional distress

The primary analyses revealed executive dysfunction as the most independent factor associated with emotional distress. Impaired attention regulation, inhibitory control, working memory, organization and goal-directed behavior may be especially important for university students who are required to attend to and control highly stimulating digital information, while also studying and having social and daily duties. The results indicate that executive-regulatory problems do not just reflect problematic short video engagement but are also informative. This interpretation is supported by the results of the simultaneous comparison of bedtime procrastination, which did not maintain an independent association, and executive dysfunction, which remained strongly associated with emotional distress. Concurrently, the high degree of correlation between the self-control deficit and executive dysfunction suggests a high overlap at conceptual and empirical levels. The results should thus not be interpreted as to indicate fully independent psychological processes. However, future research should consider the test of general self-regulatory capacity and executive dysfunction must be differentiated reliably through the confirmatory factor analysis and formal discriminant-validity test (HTMT and AVE).

### 5.3 Bedtime procrastination as a related but weaker pathway

At the same time, bedtime procrastination was positively correlated with problematic short videos engagement and emotional distress, which was consistent with previous studies that have examined how nighttime smartphone use and inability to disconnect or unwind from digital devices could affect the desired sleep patterns in university students. The fast-paced content and constant scrolling on platforms such as short videos may stand out as of particular interest because it can be hard for students to disengage if they plan to go to sleep. But the current results suggest that executive dysfunction is an important independent factor in emotional distress, such that bedtime procrastination does not best predict emotional distress. The relationship between bedtime delay and emotional vulnerability was not significant in the joint structural analysis, indicating some overlap in the link between bedtime delay and general attention, inhibition, planning and self-regulation problems. Therefore, the results cannot confirm a certain chronological order of SVA, bedtime procrastination, executive dysfunction, and emotional distress. A more defensible explanation is that these domains are related so that within the current cross-sectional data, the strongest relationship with emotional distress is between the executive dysfunction and emotional distress domains.

### 5.4 Exploratory moderation findings

#### 5.4.1 Thrill-seeking

The exploratory moderation analysis indicated that there might be a moderate interaction between SVA and executive dysfunction with thrill-seeking. The positive association between problematic short video engagement and executive dysfunction was slightly greater for those with more thrill-seeking tendencies, as the interaction between SVA and thrill-seeking was positive and statistically significant (*β* = 0.156, SE = 0.078, *p* = 0.0459). A possible explanation is that people who are more novelty-seeking, stimulated, and who prefer to see fast-changing rewards may be more sensitive to the challenges of engaging in highly stimulating short video environments. However, caution must be exercised in interpreting this finding, as the interaction was close to the conventional level of significance, it was an exploratory rather than a primary hypothesis of the study, and the effects of moderation in terms of personality must be replicated in independent samples for confirmation of stronger implications.

#### 5.4.2 Monthly living expenses

The relationship between SVA and emotional distress was relatively stable across the range of monthly living expenses – no meaningful moderation was found. This conclusion does not exclude the fact that socioeconomic conditions are not always at the center of the issue of problematic digital engagement and emotional health. Monthly living costs only give an indirect indication of socioeconomic position, and may not reflect financial stress, income, parental education, urban–rural background, or other factors related to social advantages. In future studies, therefore, socioeconomic differences in problematic short-video engagement should not be analyzed alone using costs, but rather using more comprehensive measures.

### 5.5 Theoretical implications

The results add to the body of knowledge in a variety of ways. First, they make it clear that it is essential to distinguish problematic or addictive use of short videos from short video use in general. The current results are specific to difficulty controlling engagement and its psychological correlates and should not be interpreted as an average level of engagement during watching typical short videos. In addition, the strong correlations between self-control deficit, bedtime procrastination, and executive dysfunction argue for a close link between behavioral and cognitive regulation rather than these being completely independent processes. Third, the relationship between executive dysfunction and emotional distress was significantly more independent and stronger when the two were analyzed together than when analyzed alone, when controlling for bedtime procrastination. The pattern indicates that there is a need for more emphasis on cognitive-regulatory functioning in theory-based explanations of problematic short video engagement. Since all constructs were assessed at the same time, the present study cannot conclude whether there is a causal relationship between executive dysfunction and problematic short-video engagement, whether executive dysfunction leads to problematic short-video engagement, or whether they share a vulnerability, nor can it conclude that executive dysfunction and problematic short-video engagement operate in reciprocal fashion. Longitudinal studies are thus required in the future to establish a temporal order and to test competing theoretical explanations.

### 5.6 Practical implications

In practical terms, the findings indicate that a focus on screen time alone might not be enough for digital well-being in University settings. Children who have issues with attending to short video tasks can also have focus, impulse control, organizational, sleep and emotional regulation issues. Strategies like intentional media-use planning, notification and distraction management, structured bedtime routines, self-monitoring and/or strategies to maintain goal-directed academic behavior could then be included in preventive programs. For those who are having greater issues, other cognitive, sleep-related, and emotional assessments might be more informative than simply focusing on a problem of excessive technology use. While these implications must stay guarded, the current study tested no intervention and the results are not able to prove that the changes in these factors would lead to a decrease in problematic short video engagement or emotional distress.

### 5.7 Limitations and future research

When interpreting the results, the following limitations should be taken into account. In addition, the cross-sectional design does not allow for inferences about temporal ordering and causality, so longitudinal and/or experimental studies are needed to establish whether problematic short-video engagement is a defining aspect of disorder in bedtime routines, executive functioning, and emotional well-being or if these relationships are bidirectional. Second, self-report measures were used to a large extent and could be subject to recall bias and shared-method variance, and future studies should include objective smartphone-use data, sleep data, and measures of executive functioning behavior. Third, the sample was mainly composed of female subjects and there were a large number of first-year students, which limits the generalizability and especially the geographic distribution of the sample, as it was located mainly in Henan Province. Replication, therefore, with more geographically and balanced university samples is required. Finally, there is a strong correlation between self-control deficit and executive dysfunction, so an important discriminant-validity issue has to be explored through the use of CFA-based metrics like AVE and HTMT. Finally, the structural analysis revealed mixed model fit (RMSEA = 0.125), as well as explorative moderation analyses. The structural relationships are thus tentative and not to be taken as evidence for a causal psychological mechanism. The study does not measure or directly manipulate any recommendation algorithms, so it is also not possible to determine that the design of the algorithms affected the observed behavior or emotions.

## 6 Conclusion

The results of this study indicate that the problematic short-video engagement is highly related to the self-control problem, bedtime procrastination, executive dysfunction, and emotional distress of Chinese college students. When all the regulatory factors were examined at once, it was found that executive dysfunction was the most independent in relation to emotional distress, while bedtime procrastination was less independent. The results indicate that cognitive-regulatory problems could be a significant part of the psychological profile of problematic short video engagement and that only the amount of time spent on the screen or bedtime activities might not be sufficient to capture the digital vulnerability of students. The results, however, do not provide a sequence or causal link between SVA and cognitive dysfunction and emotional distress. Caution must be used in interpreting the results due to the cross-sectional design, use of self-report measures, the geographic nature of the sample, and the overlap between some constructs, as well as poor structural-model fit. Further research is required, using longitudinal, multimethod and independently replicated designs, to elucidate the direction in which these relationships run and to establish if the executive-regulatory processes are meaningful targets for future prevention or intervention research.

## Data Availability

The data can be available on reasonable request from corresponding author.
