# Supplementary material for: How short-video usage influences college students’ daily lives: empirical evidence from sleep, self-perception, daily life conditions, and emotional state
Source: Front Psychol. 2026 Oct 2;17:1855722. doi: 10.3389/fpsyg.2026.1855722 (PMC13672586; doi:10.3389/fpsyg.2026.1855722)
Supplement: Supplementary file 1 [file Table_1.DOCX]

Dear Student,

Hello! This study aims to understand college students' media usage habits and daily life patterns. The questionnaire is anonymous, with no right or wrong answers. Responses are solely for academic research purposes. Please feel free to answer based on your actual experiences over the past month.

Special Note:

The term “short videos” in this questionnaire primarily refers to algorithm-based video apps you regularly use, such as Douyin (TikTok) and Kuaishou, which feature a “scroll-to-switch” interface. If you mainly use Douyin, please directly apply your experience of “scrolling through Douyin” when answering.

Part One: Basic Information

1. Your gender:

○ Male

○ Female

2. Your age: [Fill in] ______ years old

3. Your academic year:

Freshman

Sophomore

○ Junior

○ Senior

○ Graduate student

4. Your monthly living expenses:

A. Less than 1000 RMB

B. 1001-2000 RMB

C. 2,001–4,000 RMB

D. 4,001–6,000 RMB

E. Over 6,001 RMB

5. Average daily short video viewing time in the past week:

Under 30 minutes

30 minutes–1 hour

1–2 hours

2–3 hours

3–4 hours

Over 4 hours

Part Two: Short Video Usage Experience

Instructions: Based on your actual short video usage (e.g., TikTok) over the past week, rate how much you agree with the following statements.

(1=Never, 2=Rarely, 3=Sometimes, 4=Often, 5=Always)

Statement Options

1. I constantly think about watching short videos. 1 2 3 4 5

2. If I can't watch short videos, I feel restless or irritable. 1 2 3 4 5

3. I find myself opening the short video app unconsciously, even after just using it. 1 2 3 4 5

4. Scrolling through short videos has become a way for me to escape real-life pressures. 1 2 3 4 5

5. I often find myself spending much more time scrolling than I originally intended. 1 2 3 4 5

6. I've tried multiple times to reduce my short video viewing time, but failed each time. 1 2 3 4 5

7. Even when I have homework/tasks to complete, I can't stop swiping through videos. 1 2 3 4 5

8. I often lose track of time while scrolling through short videos. 1 2 3 4 5

9. Short video viewing takes up my sleep time, leading to insufficient rest. 1 2 3 4 5

10. My study/work efficiency has noticeably declined due to short video addiction. 1 2 3 4 5

11. Prolonged short video viewing causes discomfort in my eyes or neck. 1 2 3 4 5

12. I've neglected people around me (e.g., roommates, family) because of short videos. 1 2 3 4 5

Part Three: Sleep Habits

Instructions: Please evaluate your evening sleep habits over the past week.

(1=Almost never, 2=Rarely, 3=Sometimes, 4=Often, 5=Almost always)

Item Options

13. When it was time to sleep, I hadn't gone to bed yet. 1 2 3 4 5

14. I want to go to bed early at night but always fail to do so. 1 2 3 4 5

15. When it's time to sleep, I am easily distracted by other things (e.g., my phone). 1 2 3 4 5

16. I often waste time doing unimportant things at night instead of going to sleep. 1 2 3 4 5

17. I frequently stay up late for no particular reason. 1 2 3 4 5

18. My actual bedtime is later than I intended. 1 2 3 4 5

Part IV: Self-Description

Instructions: Please indicate whether the following statements describe you typically.

(1=Does not describe me at all, 2=Does not describe me much, 3=Describes me somewhat, 4=Describes me fairly well, 5=Describes me completely)

Statement Options

19. I find it hard to break bad habits. 1 2 3 4 5

20. I am lazy. 1 2 3 4 5

21. I sometimes speak inappropriately (tendency to speak impulsively). 1 2 3 4 5

22. I do certain things purely for the thrill, even if they are detrimental to me. 1 2 3 4 5

23. To ensure you're reading carefully, please select “Not at all like me” for this question. (Lie-detector question) 1 2 3 4 5

24. I find it hard to concentrate. 1 2 3 4 5

25. I can be very patient to get what I want.1 2 3 4 5

26. People consider me to have strong self-control. 1 2 3 4 5

Part V: Daily Life Conditions

Instructions: Please indicate whether you have experienced the following situations in the past month.

(1=Never, 2=Rarely, 3=Sometimes, 4=Often, 5=Always)

Item Option

(Inhibition Control Dimension)

27. I tend to act impulsively without considering the consequences. 1 2 3 4 5

28. When I experience negative emotions, I tend to overreact. 1 2 3 4 5

29. I frequently interrupt others when they are speaking. 1 2 3 4 5

30. I find it difficult to stop what I'm doing at the appropriate time. 1 2 3 4 5

31. I make rash decisions that I later regret. 1 2 3 4 5

32. Even when I know I shouldn't say something, I can't help but blurt it out. 1 2 3 4 5

(Working Memory Dimension)

33. I often forget where I was putting something just moments ago. 1 2 3 4 5

34. I struggle to remember instructions given just minutes ago. 1 2 3 4 5

35. When reading, I often forget the beginning after reaching the end. 1 2 3 4 5

36. During complex tasks, I easily lose track of my progress. 1 2 3 4 5

37. I find it difficult to process multiple pieces of information simultaneously. 1 2 3 4 5

38. Even when I try hard to remember, I still forget things easily. 1 2 3 4 5

(Cognitive Flexibility Dimension)

39. I find it difficult to adapt to sudden changes in plans. 1 2 3 4 5

40. I get irritated when things don't go as expected. 1 2 3 4 5

41. My thoughts often get stuck on a single problem without progress. 1 2 3 4 5

42. I tend to rely on old methods to solve problems, even when they don't work. 1 2 3 4 5

43. Switching from one task to another is difficult for me. 1 2 3 4 5

44. My thinking lacks flexibility. 1 2 3 4 5

Part Six: Recent Emotional State

Instructions: Based on your actual feelings over the past week, select the option that best describes you.

(1=Never, 2=Rarely, 3=Sometimes, 4=Often, 5=Always)

Item Option

45. I find it hard to calm myself down. 1 2 3 4 5

46. I feel dry mouth and throat. 1 2 3 4 5

47. I seem to have no positive feelings at all. 1 2 3 4 5

48. I experience difficulty breathing (e.g., wheezing or feeling short of breath). 1 2 3 4 5

49. I find it hard to start studying or working. 1 2 3 4 5

50. I experience physical tremors (e.g., shaking hands) even without strenuous activity. 1 2 3 4 5

51. I often overreact to situations. 1 2 3 4 5

52. I feel there's little to look forward to these days. 1 2 3 4 5

53. I easily feel tense. 1 2 3 4 5

54. I feel downcast and melancholy. 1 2 3 4 5

55. I've lost confidence in myself. 1 2 3 4 5

56. I easily feel panicked. 1 2 3 4 5

57. I find no interest in anything.1 2 3 4 5

58. I feel life lacks meaning. 1 2 3 4 5
